# Inhibitory Effects of Cenobamate on Multiple Human Cardiac Ion Channels and Possible Arrhythmogenic Consequences

**DOI:** 10.3390/biom14121582

**Published:** 2024-12-11

**Authors:** Andreea Larisa Mateias, Florian Armasescu, Bogdan Amuzescu, Alexandru Dan Corlan, Beatrice Mihaela Radu

**Affiliations:** 1 Department of Anatomy, Animal Physiology and Biophysics, Faculty of Biology, University of Bucharest, Splaiul Independentei 91-95, 050095 Bucharest, Romania; andreealarisa.mateias@studenti.univr.it (A.L.M.); florianarmasescu@gmail.com (F.A.); beatrice.radu@bio.unibuc.ro (B.M.R.); 2 Department of Biotechnology, University of Verona, Strada Le Grazie 15, 37134 Verona, Italy; 3 Cardiology Research Unit, University and Emergency Hospital of Bucharest, Splaiul Independenței 169, 050098 Bucharest, Romania; alexandru@corlan.net

**Keywords:** cardiac Na^+^ channel, cenobamate, class Ib antiarrhythmic, reentry arrhythmia, hiPSC-CM, ventricular cardiomyocyte tissue model, whole-cell patch-clamp

## Abstract

Cenobamate is a novel third-generation antiepileptic drug used for the treatment of focal onset seizures and particularly for multi-drug-resistant epilepsy; it acts on multiple targets: GABA_A_ receptors (EC_50_ 42–194 µM) and persistent neuronal Na^+^ currents (IC_50_ 59 µM). Side effects include QT_c_ interval shortening with >20 ms, but not <300 ms. Our in vitro cardiac safety pharmacology study was performed via whole-cell patch-clamp on HEK293T cells with persistent/inducible expression of human cardiac ion channel isoforms hNav1.5 (*I*_Na_), hCav1.2 (α1c + β2 + α2δ1) (*I*_CaL_), hKv7.1 + minK (*I*_Ks_), and hKv11.1 (hERG) (*I*_Kr_). We found IC50 of 87.6 µM (peak *I*_Na_), 46.5 µM (late *I*_Na_), and 509.75 µM (*I*_CaL_). In experiments on Ncyte^®^ ventricular cardiomyocytes, APD90 was reduced with 28.6 ± 13.5% (mean ± SD) by cenobamate 200 µM. Cenobamate’s marked inhibition of *I*_Na_ raises the theoretical possibility of cardiac arrhythmia induction at therapeutic concentrations in the context of preexisting myocardial pathology, in the presence of action potential conduction and repolarization heterogeneity. This hypothetical mechanism is consistent with the known effects of class Ib antiarrhythmics. In simulations with a linear strand of 50 cardiomyocytes with variable inter-myocyte conductance based on a modified O’Hara–Rudy model, we found a negligible cenobamate-induced conduction delay in normal tissue, but a marked delay and also a block when gap junction conduction was already depressed.

## 1. Introduction

Cenobamate (formerly known as YKP3089) is a novel antiepileptic drug that was approved by the US Food and Drug Administration in November 2019 and by the European Medicines Agency in March 2021. It is commercialized in the US by SK Life Science Inc. and in Europe by Arvelle Therapeutics Netherlands BV under the commercial names Xcopri^®^ and Ontozry^®^, respectively [1]. The drug has been successfully used for treating adults with focal onset seizures and particularly for epilepsy resistant to other antiepileptic drugs, achieving a reduction in monthly seizure frequency of up to 55% and unprecedented seizure-free rates of up to 28% [2]. Cenobamate is a tetrazole alkyl carbamate derivative featuring some structural similarity to earlier compounds such as meprobamate, felbamate, carisbamate, and retigabine. Interestingly, cenobamate was discovered initially via a purely phenotypic screening; its main pharmacodynamic effects were identified later and they are still incompletely understood [3]. Its impressive effectiveness is due to action on multiple targets, including positive allosteric modulatory effects on GABA_A_ receptors by binding to a site different from the benzodiazepine binding site [4]. GABA_A_ receptor binding exerts limited agonistic effects and occurs with different affinities for the six α subunit isoforms tested, α1β2γ2 and α2-6β3γ2, with reported EC_50_ values of 42 to 194 µM ([5] pp. 8–9). The effects on GABA_A_ receptors are somewhat similar to those of barbituric compounds, leading to both tonic and phasic GABA_A_-mediated inhibition [1]. Another essential contribution to the antiseizure potency of cenobamate is provided by its inhibitory effects on neuronal Na^+^ channels, particularly on the late (persistent) Na^+^ current component (reported IC_50_ of 59 µM) [6]. This selective inhibition of the persistent neuronal Na^+^ current component may explain the relatively specific inhibition of excitatory pyramidal neuron activity without significant effects on inhibitory interneurons [7]. However, the complex pharmacokinetics of the compound pose some difficulties, requiring careful titration and maintenance, with starting doses of 12.5 mg/day increased gradually to a recommended dose of 200 mg/day and maximally 400 mg/day, where effective peak plasma therapeutic concentrations (*C*_max_) reach 45.5 µg/mL (169.98 µM), and the average binding to plasma proteins is ~60% (study YKP3089C018) ([5] p. 13). Beyond common side effects such as dizziness, drowsiness, headache [8], and an increased tendency to suicide, cenobamate reduces the QTc interval by more than 20 ms, although not below 300 ms; therefore, it is forbidden for patients with hereditary short QT syndrome. These QT shortening effects were assumed to result from the inhibition of cardiac voltage-dependent Na^+^ channels and further post-marketing studies were recommended ([9] pp. 11–12). Therefore, the aim of our study was to provide an in-depth evaluation of cenobamate’s effects on different human cardiac ion channels via in vitro patch-clamp experiments according to the principles of the Comprehensive in vitro Proarrhythmia Assay (CiPA) paradigm [10] and to perform a preliminary exploration, via computational approaches, of the possible proarrhythmogenic consequences of clinical relevance.

## 2. Materials and Methods

### 2.1. Cell Cultures

Experiments were performed on HEK293T cell lines with persistent/inducible expression of different human cardiac ion channel isoforms: hNav1.5 and hKv7.1 + minK (Anaxon AG, Bern, Switzerland), hCav1.2 (α1c + β2 + α2δ1) (B’SYS GmbH, Witterswil, Switzerland), and hKv11.1 (hERG) (Cytocentrics Bioscience GmbH, Rostock, Germany). The cell lines were grown in standard conditions (37 °C, 5% CO_2_, 90% humidity) in DMEM:F12 medium (D8900, Sigma-Aldrich, Burlington, MA, USA) buffered with 15 mM HEPES, containing 3.15 g/L D-glucose and 0.055 g/L sodium pyruvate, supplemented with 1.2 g/L sodium bicarbonate, 10% fetal bovine serum (F2442, Sigma-Aldrich, Burlington, MA, USA), 2 mM L-glutamine (G7513, Sigma-Aldrich, Burlington, MA, USA), 1% penicillin–streptomycin (P4333, Sigma-Aldrich, Burlington, MA, USA), and with specific antibiotics for selection of transfected cells or induction of gene expression according to manufacturers’ instructions. The medium was changed every 2–3 days and the cells were passed weekly upon detachment with trypsin–EDTA solution. All cell culture media and solutions were sterile filtered with 0.22 μm polyvinylidene fluoride filters. Ncyte^®^ vCardiomyocytes (Ncardia, Leiden, The Netherlands) were cultured in 24-well plates precoated with bovine fibronectin, following manufacturer’s instructions. For the experiments, cardiomyocytes were detached as described previously [11] and plated on ϕ 10 mm glass coverslips, like the other cell lines.

### 2.2. Whole-Cell Patch-Clamp Pharmacology Assays

Manual whole-cell patch-clamp experiments were performed at room temperature, with equipment as described in previous studies [11,12]. Pipettes were pulled from Clark borosilicate glass capillaries with inner filament (GC150F-10, Harvard Apparatus, Holliston, MA, USA) and had typical resistances of 2–3 MΩ. Recordings were performed with a WPC-100 resistive feedback amplifier (ESF electronic, Göttingen, Germany) or an Axopatch 200B amplifier (Axon Instruments—Molecular Devices, San Jose, CA, USA) connected to a Digidata 1322A interface driven by the pClamp 8.2 Clampex module (Axon Instruments, part of Molecular Devices, San Jose, CA, USA). For different types of ion channel, we applied specific voltage-clamp protocols as follows:

- For hERG, a standard two-step activation protocol was used: holding at −70 mV, a 100 ms step at −50 mV used as current reference for measurements, a 2 s depolarizing step at +40 mV, followed by a 2 s step at −50 mV to elicit the peak hERG current by rapid recovery from inactivation, repeated sweeps at 10 s intervals, plus specific protocols to assess the voltage dependence of activation and inactivation, as described in Thomet et al., 2021 [11].
- For hKv7.1 + minK, a general two-step activation protocol was used: holding at −110 mV, a 250 ms step at −80 mV with a brief (50 ms) step at −90 mV at the middle to monitor seal quality, a 3000 ms depolarizing step at +60 mV, followed by a 1000 ms step at −50 mV; repeated sweeps at 20 s intervals.
- For hCav1.2, a pharmacology protocol with holding at −80 mV, a first 100 ms step at −50 mV and a second 200 ms step at 0 mV, repeated sweeps at 20 s intervals with drug application after 5 sweeps if current levels were steady, plus an activation–inactivation protocol with holding at −80 mV, 1000 ms depolarizing steps from −80 to +50 mV in 10 mV increments, followed by 250 ms steps at +10 mV and a return to −80 mV; 14 sweeps at 20 s intervals.
- For hNav1.5, multiple voltage protocols were used, including the following:
- A standard pharmacology protocol with holding at −90 mV, a 20 ms step at −110 mV for seal monitoring, a 40 ms activation step at −10 mV, and repeated sweeps at 1 s intervals. Initially, 60 sweeps were recorded in control conditions, and 30 sweeps at 3 min after drug or vehicle solution application.
- An activation protocol with holding at −90 mV, a 20 ms step at −110 mV for seal monitoring, depolarizing 40 ms steps from −80 mV to +60 mV in 5 mV increments, followed by a return to −90 mV; repeated sweeps at 5 s intervals.
- A steady-state inactivation protocol with holding at −120 mV, 1000 ms activation steps from −120 mV to +40 mV, followed by a 25 ms step at −30 mV; repeated sweeps at 5 s intervals.
- A use-dependent block protocol with holding at −120 mV and 5 depolarizing pulses at −10 mV lasting 10 ms each, with interpulse intervals increasing from sweep to sweep from 5 ms to 50 ms in 5 ms increments.
- A CiPA-like protocol for experiments with ATX-II to measure effects on late Na^+^ current, similar to that described in [11], except for a holding potential of −90 mV, a preconditioning step at −110 mV, and a repeat interval of 8 s.

Experiments on Ncyte^®^ vCardiomyocytes were performed via an amphotericin-perforated whole-cell approach, filling the pipettes with solution containing amphotericin B 300 µg/mL except for a small region near the tip that contained amphotericin-free solution. The voltage-clamp and current-clamp protocols were similar to those used in our previous studies [11,12,13]; the following protocols were recorded in sequence before and at 3 min after application of cenobamate in the bath to a final concentration of 200 µM:

- Nav standard protocol: holding −90 mV, depolarizing step at −10 mV lasting 40 ms;
- Cav-Nav separation protocol: holding −80 mV, first step at −20 mV 200 ms, intermediate step at −40 mV 100 ms, second step at −50 mV to +50 mV 200 ms, in 10 mV increments in consecutive sweeps;
- hERG standard protocol: holding −70 mV, a brief preliminary step at −50 mV 100 ms, first step at +40 mV 2000 ms, second step at −120 mV/−50 mV 2000 ms (in consecutive sweeps);
- A double voltage ramp protocol: holding −80 mV, first ramp from −120 mV to +80 mV in 2000 ms, second ramp from +80 mV back to −120 mV in 2000 ms, sweeps repeated at 10 s intervals;
- A current-clamp protocol with external pacing: 3 current stimuli of 2 nA amplitude, duration 0.5 ms (up to 2.5 ms in some experiments) applied at 3 s intervals, first stimulus at 500 ms after start of recording; in cells with spontaneous pace-making, this phenomenon was suppressed by applying a steady hyperpolarizing current of −10 pA or −20 pA.

For experiments, cells were detached with trypsin-EDTA, centrifuged, resuspended in standard extracellular solution, and placed in 24-well culture plates containing ϕ 10 mm glass coverslips sterilized by brief immersion in pure ethanol and soaking. After 30 min incubation at 37 °C to allow cell attachment, each coverslip was removed from the well and placed at the center of a ϕ 35 mm Petri dish, surrounded by a Teflon™ ring with a central hole of the same diameter as the coverslip, with the inner edge covered with silicone grease (Merck KGaA 1.07746.0100, Rahway, NJ, USA). After pressing the Teflon™ ring to create a water-tight seal, 100 μL of standard extracellular solution was pipetted over the coverslip. During patch-clamp experiments, 10 μL of cenobamate intermediate solution in extracellular solution at 10× the desired final concentration was added, approximately compensating for the solution loss by evaporation during the time since mounting the coverslip until drug application.

### 2.3. Chemicals and Solutions

In all experiments, the standard extracellular solution had the following composition (in mM): NaCl 140, KCl 2.5, MgCl_2_ 2, CaCl_2_ 2, HEPES 10, D-glucose 10, sucrose 15, pH 7.40 at 25 °C titrated with NaOH 1 M. In experiments performed on hKv11.1 (hERG) channels, the pipette solution contained (in mM): KCl 140, CaCl_2_ 1, MgCl_2_ 2, EGTA 11, HEPES 10, pH 7.20 at 25 °C titrated with KOH 1 M. In experiments on hKv7.1 + minK, this solution was supplemented with MgATP 5 mM, and in later experiments also with creatine phosphokinase (238395, EMD Millipore, Burlington, MA, USA) at 50 U/mL final concentration and phorbol 12-myristate 13-acetate (PMA, 524400, EMD Millipore, Burlington, MA, USA) freshly added from a 10 mM stock solution in DMSO to a final concentration of 10 μM. For experiments on hNav1.5 and hCav1.2, the pipette solution contained (in mM): D-gluconic acid 70, CsCl 60, MgCl_2_ 5, CaCl_2_ 1, Na_2_ATP 5, EGTA 11, HEPES 10, pH 7.20 by titration with CsOH 1 M. For experiments on Ncyte^®^ vCardiomyocytes, the pipette solution contained (in mM): K^+^ gluconate 100, KCl 20, CaCl_2_ 1, MgCl_2_ 1, EGTA 11, HEPES 10, Na_2_phosphocreatine 3, MgATP 4, pH 7.20 at 25 °C titrated with KOH. Amphotericin B (E3789, Sigma, Burlington, MA, USA) was used to prepare a stock solution with a concentration of 30 mg/dL, kept at −20 °C and protected from light. ATX-II (STA-700, Alomone labs, Jerusalem, Israel) was dissolved in pure water to obtain a 20 µM stock solution; aliquots were stored at −20 °C and used to prepare the working solution at 20 nM or 100 nM in extracellular solution. A primary 200 mM stock solution of cenobamate (purchased from Selleckchem, Houston, TX, USA or DC Chemicals, Shanghai, China) in DMSO was stored at −80 °C for several months, with small samples stored at −20 °C for experiments for less than 1 month. In a large number of experiments, including those on hiPSC-CM, the final estimated concentration in the recording chamber was 200 μM, which is slightly higher that the reported maximal plasma therapeutic effective concentration in human trials for a dose of 400 mg/day (*C*_max_ = 169.98 μM, [5] p. 10). In each whole-cell patch-clamp experiment, the same sequence of voltage protocols was applied initially and upon drug exposure, and the effect on peak or plateau current levels was assessed when a steady state was reached.

### 2.4. Data Analysis and Computer Simulations

Throughout the paper, data are reported as mean ± SD or mean ± SEM, as appropriate, with *n* indicating the number of cells recorded in each experimental condition. Statistical significance was tested via Student’s *t* test or non-parametric variants if the normality condition was not met, using a critical level of *α* = 0.05. Computer simulations at single-cardiomyocyte level or on a 1D linear string model with *n* = 50 cells were developed using C++ scripts and R scripts for postprocessing and visualization (GCC 11.4.0 and R 4.1.2 from the Linux Mint v21.3 distribution), starting from a previously published version of a modified O’Hara–Rudy 2011 human ventricular cardiomyocyte electrophysiology model [14] optimized for pharmacology assays [11], by including cardiac ion channel inhibition data and blocking/unblocking kinetics specific for cenobamate, and considering different gap junction conductance values that are specific for normal or pathological intercellular coupling in the working ventricular myocardium.

## 3. Results

### 3.1. Assessment of Inhibitory Effects of Cenobamate on Different Cardiac Ion Channels and Action Potentials Recorded in hiPSC Cardiomyocytes

**Figure 1:**
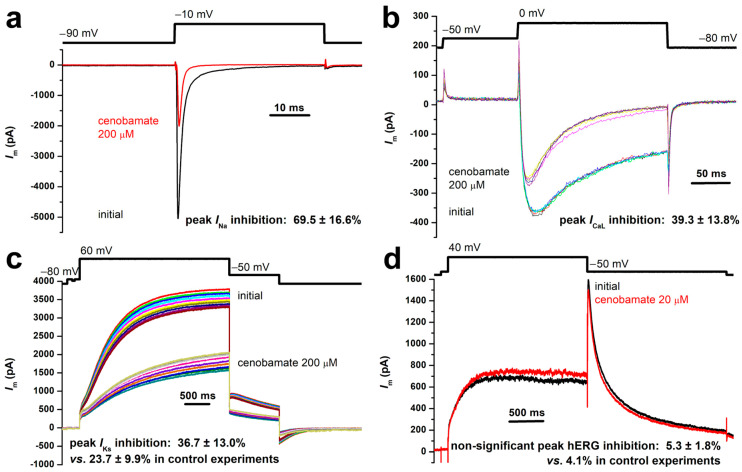
Inhibitory effects of cenobamate on multiple human cardiac ion channels. (**a**) Effect on *I*_Na_ assessed with a standard voltage protocol; (**b**) effect on *I*_CaL_; (**c**) effect on *I*_Ks_; and (**d**) effect on *I*_Kr_ (hERG channels).

Using the experimental settings and protocols described in Section 2, we performed in vitro assays to test the inhibitory effects of cenobamate on multiple human cardiac ion channels expressed in HEK293T cells. Within an initial set of experiments, the drug was applied at a final concentration of 200 μM. Thus, we measured an average inhibition of peak *I*_Na_ elicited with the standard protocol of 69.53 ± 14.63% (mean ± SD, *n* = 6 experiments) vs. 0.78 ± 1.82% in control experiments (*n* = 5) where only the solvent (DMSO) was applied in similar conditions at an equivalent final concentration. Individual results are listed in Table S1 of the Supplementary Materials, and the inhibition in a typical experiment is illustrated in Figure 1a. With these experimental data, we computed an apparent IC50 value for peak *I*_Na_ inhibition by cenobamate of 87.64 μM.

**Figure 2:**
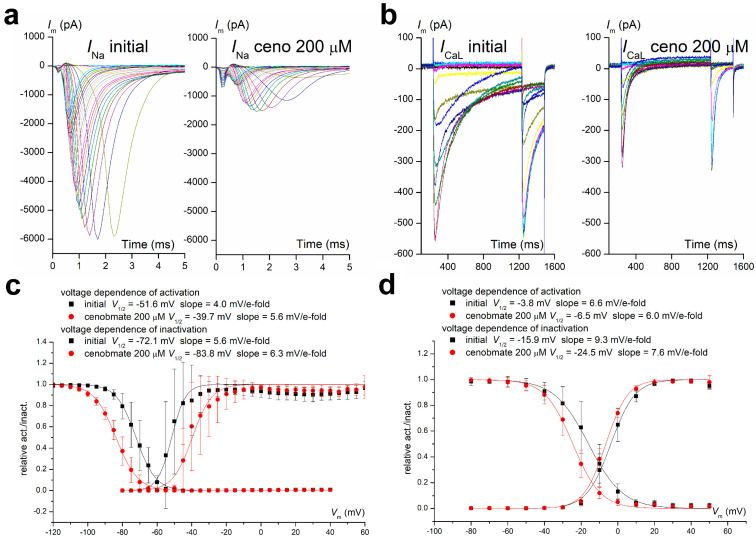
Effects of cenobamate on voltage dependence of activation and steady-state inactivation of several cardiac ion channels. (**a**) hNav1.5 *I*_Na_ elicited by voltage steps from −80 mV to +60 mV in 5 mV increments initially and at 3 min after application of cenobamate 200 µM; (**b**) hCav1.2 *I*_CaL_ recorded during activation steps from −80 mV to +50 mV followed by a step at +10 mV initially and at 3 min after application of cenobamate 200 µM; (**c**) voltage shifts of activation and inactivation of hNav1.5; (**d**) voltage shift of hCav1.2 inactivation. Data are mean values of *n* = 6 experiments for *I*_Na_ and *n* = 4 experiments for *I*_CaL_; error bars represent SD.

**Figure 3:**
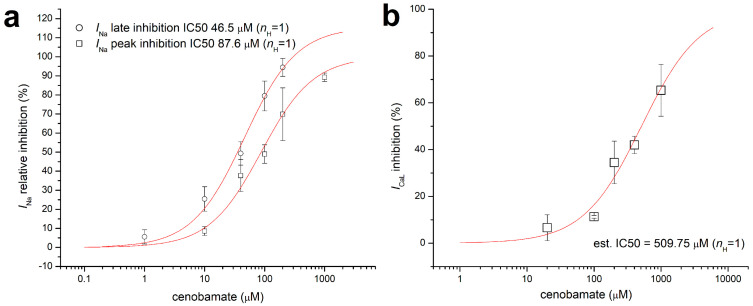
Effects of cenobamate on *I*_Na_ and *I*_CaL_. (**a**) Dose–response curves of cenobamate inhibition of hNav1.5 peak and late current, fitted with Hill functions with unitary Hill coefficient (*n*_H_ = 1); (**b**) dose–response curves of cenobamate inhibition of hCav1.2 peak current measured during the depolarizing step at 0 mV. Error bars represent SD (see Tables S2 and S3 of Supplementary Materials).

Furthermore, we evidenced voltage shifts larger than 10 mV for both the voltage dependence of activation and steady-state inactivation, as shown in Figure 2c, where data points were fitted with Boltzmann charge-voltage functions; a typical experiment with recordings of *I*_Na_ activated at multiple voltage steps initially and in the presence of cenobamate 200 µM is shown in Figure 2a. We also found that the inhibition by cenobamate of peak *I*_Na_ is virtually voltage-independent, by dividing peak *I*_Na_ amplitude for multiple depolarizing voltage steps in the presence of cenobamate by the initial amplitude for equivalent voltage steps in a recording performed before applying the blocker, as illustrated in Figure S1 of the Supplementary Materials. Such a behavior is to be expected for a drug with a neutral molecule at physiological pH. In a subsequent set of experiments, we tested the effects of multiple cenobamate concentrations on the peak Na^+^ current, assessed with a standard voltage protocol with a single depolarizing step at −10 mV, and on the late Na^+^ current, assessed with a CiPA-like voltage protocol upon adding ATX-II 20 nM or 100 nM in the bath solution. Typical recordings in this setting are illustrated in Figure S2 of the Supplementary Materials, and experimental measurements are listed in Tables S2 and S3 of the Supplementary Materials. The dose–response curves and apparent IC50 values for cenobamate inhibition of peak and late *I*_Na_ (87.6 µM and 46.5 µM, respectively) are illustrated in Figure 3a.

Within an initial set of experiments on L-type Ca^2+^ channels, we obtained an average inhibition of peak *I*_CaL_ amplitude elicited with the standard protocol during the depolarizing step at 0 mV of 39.26 ± 13.78% (*n* = 4) vs. 3.10 ± 2.89% in control experiments (*n* = 3), as shown in Figure 1b and Table S4 of the Supplementary Materials. We also demonstrated a cenobamate-induced voltage shift, particularly of the relative steady-state inactivation, as shown in Figure 2d. A typical experiment with recordings of *I*_CaL_ activated at multiple voltage steps between −80 mV and +50 mV, followed by a step at +10 mV, initially and in the presence of cenobamate 200 µM, is shown in Figure 2b. We repeated the experiments at multiple cenobamate concentrations (results listed in Table S5 of the Supplementary Materials), obtaining an apparent IC50 value for hCav1.2 inhibition by cenobamate of 509.75 µM by using a dose–response function with unitary Hill coefficient, as shown in Figure 3b.

For *I*_Ks_, the average inhibition of the plateau current at +60 mV was 36.68 ± 13.02% (*n* = 8) vs. 23.66 ± 9.86% in control experiments (*n* = 6), as shown in Figure 1c and Table S6 of the Supplementary Materials. Thus, the estimated net average inhibition (cenobamate 200 μM—control) was 36.68 − 23.66 = 13.02%, resulting in an estimated IC50 for *I*_Ks_ (Kv7.1 + minK) of 1336.1 μM.

Finally, in experiments on hERG channels, we obtained an average peak *I*_Kr_ inhibition by cenobamate 20 μM of 5.3 ± 1.8% vs. 4.1% in control experiments, as shown in Figure 1d. Subsequent experiments on hERG channels performed with a cenobamate concentration of 200 µM provided a relative peak *I*_Kr_ inhibition of 10.97 ± 3.11% (Table S7 of Supplementary Materials). With these data, we computed an average peak *I*_Kr_ inhibition at 200 µM cenobamate of 10.97 − 4.1 = 6.87%, resulting in an estimated IC_50_ of 2711.2 µM. However, we preferred to consider for further computations the apparent cenobamate IC50 for *I*_Kr_ (hERG) of 1869 μM reported in preliminary studies required for drug approval ([5] p. 10).

**Figure 4:**
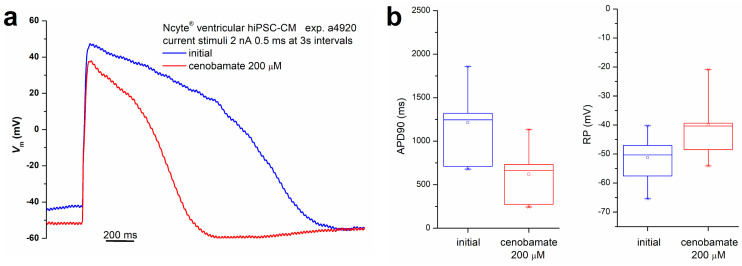
Effects of cenobamate 200 µM on AP shape and parameters. (**a**) Current-clamp recordings showing the AP triggered by an external current stimulus (2 nA, 0.5 ms) in control conditions and during drug application in a typical experiment; (**b**) distribution of APD90 and RP (initial values and during application of cenobamate 200 µM) in experiments performed on Ncyte^®^ vCardiomyocytes hiPSC-CM.

We also performed a series of amphotericin-perforated whole-cell experiments on Ncyte^®^ vCardiomyocytes hiPSC-CM preparations. In this setting, cenobamate 200 µM induced an average APD90 shortening by 28.6 ± 13.5% (mean ± SD, *n* = 5) and a resting potential (RP) depolarization from −51.3 ± 7.8 mV (*n* = 8) to −40.1 ± 10.4 mV (*n* = 7). The results are listed in Table S8 of Supplementary Materials, while a typical current-clamp recording showing external stimulus-triggered action potentials (APs) and the distribution of AP duration (APD90) and RP values are illustrated in Figure 4a,b.

### 3.2. Estimation of State-Dependent Blocking and Unblocking Rates of Cenobamate for Nav1.5 Channels

**Figure 5:**
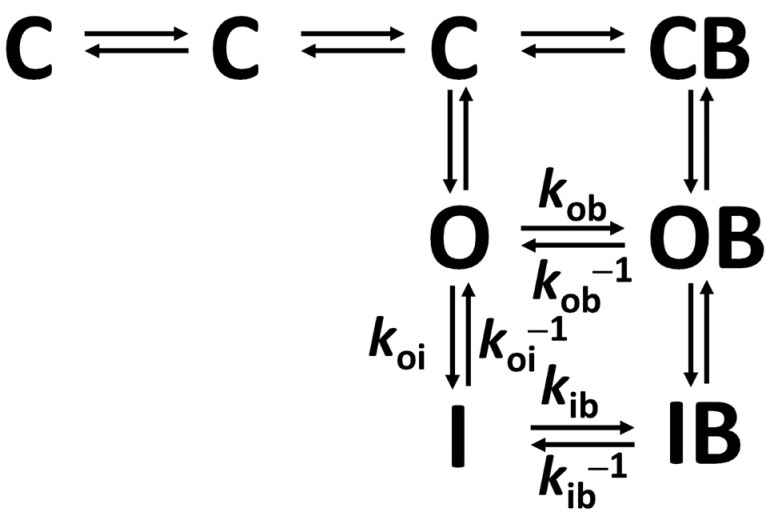
Markov state diagram of Nav1.5 channels gating, including drug block according to the MRH.

In this approach, we adopted the modulated receptor hypothesis (MRH) of Na^+^ channel blocks by local anesthetics [15,16] for our extended Markov state diagram of Nav1.5 channel gating, including conformation-specific blocked states, as illustrated in Figure 5.

To estimate state-specific blocking and unblocking kinetics, we applied, initially and during exposure to cenobamate, a special voltage protocol devised for use-dependent block, with repeated brief depolarizing pulses at −10 mV separated by variable intervals at a hyperpolarized potential (−120 mV), with durations between 5 and 50 ms, as described in Section 2. Recordings using this protocol in initial conditions and with cenobamate application are illustrated in Figure S3 of the Supplementary Materials. By noting with *t_d_* = 10 ms and *t_r_* = 5–50 ms the times spent at a depolarized potential and at a resting hyperpolarized potential during each pulse, with *o_n_* the fraction of available (non-inactivated or blocked) channels that produce the peak Na^+^ current during each depolarizing pulse of the sequence, and with *τ_i_* and *τ_r_*, the apparent macroscopic time constants of inactivation and recovery from inactivation in control conditions (in the absence of a blocker), we obtained using several approximations a general recurrence formula that allows computation for each experiment of the recovery time constant, knowing the inactivation time constant (obtained by the monoexponential fit of the inactivation part of the recording during the depolarization pulse) and the decay in peak *I*_Na_ amplitude during pulse *n* relative to the peak amplitude during the preceding pulse (1 − *o_n_*)/*o_n_*_−1_ (detailed explanations in Section S1 of the Supplementary Materials):

$$\tau_{r}=t_{r}/\left[\ln\left(1-e^{-t_{d}/\tau_{i}}\right)-\ln\left(\frac{1-o_{n}}{o_{n-1}}\right)\right]$$

Furthermore, for application of the same voltage protocol during exposure to cenobamate, by using a similar reasoning and considering a composed “inactivated-or-blocked” state, we obtained a formula for computing an estimate of the blocking/unblocking time constant specific for the inactivated conformation of the channels (*τ_ib_*) starting from *τ_i_* and an apparent time constant for open channel blocks (*τ_ob_*):

$$\tau_{ib}=1/\left(1/\tau_{r}-\left[\ln\left(1-e^{-t_{d}(1/\tau_{i}+1/\tau_{ob})}\right)-\ln\left(\frac{1-o_{n}}{o_{n-1}}\right)\right]/t_{r}\right)$$

Using these computation methods, we obtained by analyzing data obtained in four experiments performed on hNav1.5-expressing HEK293 cells, with application of a voltage-clamp protocol including five depolarizing pulses (−10 mV, *t_d_* = 10 ms) with interpulse intervals at −120 mV increasing from 5 to 50 ms in 5 ms increments (*t_r_* = 5–50 ms), an estimated *τ_ib_* of 4.03 ± 0.70 ms (mean ± SD, see Table 1). Starting from this estimated value, we computed *k*_ib_^−1^ and *k*_ib_, assuming that their ratio (the inactivated-specific *K*_d_) should follow the same proportion relative to the open-specific *K*_d_, as in the case of lidocaine-Nav1.5, estimated from the experimental data of Cardona K et al., 2010 [17]:

*k*_ob_ = 5173 M^−1^ ms^−1^, *k*_ib_ = 4998.4 M^−1^ ms^−1^, *k*_ob_^−1^ = 0.0128 ms^−1^, *k*_ib_^−1^ = 0.0384 ms^−1^

*K*_d_ (I) = *k*_ib_^−1^/*k*_ib_ = 0.0384 ms^−1^/0.0049984 µM^−1^ ms^−1^ = 7.6825 µM

*K*_d_ (O) = *k*_ob_^−1^/*k*_ob_ = 0.0128 ms^−1^/0.005173 µM^−1^ ms^−1^ = 2.4744 µM

*K*_d_ (I)/*K*_d_ (O) = 3.1048

For cenobamate, using the same state-specific dissociation constant ratio, and noting with [*D*] the drug (cenobamate) concentration used in experiments (estimated to 98.44 µM based on apparent inhibition of peak *I*_Na_ amplitude)

*K*_d_ (O) = 87.77 µM; *K*_d_ (I) = 87.77 µM × 3.1048 = 272.5083 µM

*k*_ib_ × [*D*] + *k*_ib_^−1^ = 1/*τ_ib_* = 0.2484 ms^−1^ => 0.2484 = (*k*_ib_^−1^/272.5083) × 98.44 + *k*_ib_^−1^

*k*_ib_^−1^ = 0.2484/(1 + 98.44/272.5083) = 0.18252 ms^−1^

*k*_ib_ = (0.2484 − 0.18252)/98.44 = 0.06593 ms^−1^/98.44 µM = 0.0006698 µM^−1^ ms^−1^

To estimate the blocking and unblocking rates specific for the open channel conformation (*k*_ob_ and *k*_ob_^−1^), we assessed the apparent macroscopic time constant of current decay via the monoexponential fitting of the inactivation phase of *I*_Na_ elicited with the standard Nav voltage protocol in the same cell before and during the addition of cenobamate 200 µM. Thus, we obtained, in drug exposure and control experiments, the time constants presented in Table 2. With these kinetic data, we directly estimated the two rates, by making a supplementary assumption: the dissociation constant (*K*_d_) is equivalent to the IC_50_ of cenobamate on peak *I*_Na_ amplitude (87.8 µM). Consequently, we obtained the system of two algebraic equations:

*K*_d_ = *k*_ob_^−1^/*k*_ob_ = 87.77 µM  200 µM × *k*_ob_ + *k*_ob_^−1^ = 0.62 ms^−1^

leading to:

*k*_ob_ = 0.62/287.77 = 0.00215 µM^−1^ ms^−1^  *k*_ob_^−1^ = 0.189 ms^−1^

### 3.3. Inclusion of Pharmacological Inhibition Experimental Data in a Ventricular Cardiomyocyte Electrophysiology Model: Testing Effects on Action Potential and Computing Proarrhythmogenic Risk Predictors

We applied all the experimental inhibition data and state-dependent blocking kinetics for Nav1.5 to a modified O’Hara–Rudy 2011 model adapted for pharmacology simulations and specifically for computing the proarrhythmogenic risk predictor *Q*_net_, as described in [18,19]. The starting C++ script, described in [11] (available on GitHub at https://github.com/bamuzesc/Qnet-for-CiPA.git and on Zenodo at https://zenodo.org/record/5615548#.YXvbQ7hyGSp, accessed on 10 October 2024), has been completed by adding pharmacological inhibition data for the newly defined compound cenobamate, and the state-specific Nav1.5 blocking–unblocking rates have been inserted in the Hodgkin–Huxley-type gating model of *I*_Na_ defined in the subroutine RGC (rates, gates, and currents), as described in Section S2 of the Supplementary Materials. These changes were performed according to the method used by Cardona et al., 2010 [17], by defining a blocked state probability *b* and expressing its rate of change by a first order differential equation:

$$\begin{array}{c} db/dt=\left\{m^{3}\left[(1-f_{I_{NaP}})hj+f_{I_{NaP}}h_{p}j_{p}\right]k_{ob}+m^{3}\left[1-(1-f_{I_{NaP}})hj-f_{I_{NaP}}h_{p}j_{p}\right]k_{ib}\right\}[D](1-b)-\\ -\left\{m^{3}\left[(1-f_{I_{NaP}})hj+f_{I_{NaP}}h_{p}j_{p}\right]k_{ob}^{-1}+m^{3}\left[1-(1-f_{I_{NaP}})hj-f_{I_{NaP}}h_{p}j_{p}\right]k_{ib}^{-1}\right\}b \end{array}$$

and inserting its value, computed by numeric integration, in the current equation:

$$I_{Na}=(1-b)G_{Na}(V-E_{Na})m^{3}\left[(1-f_{I_{NaP}})hj+f_{I_{NaP}}h_{p}j_{p}\right]$$

**Figure 6:**
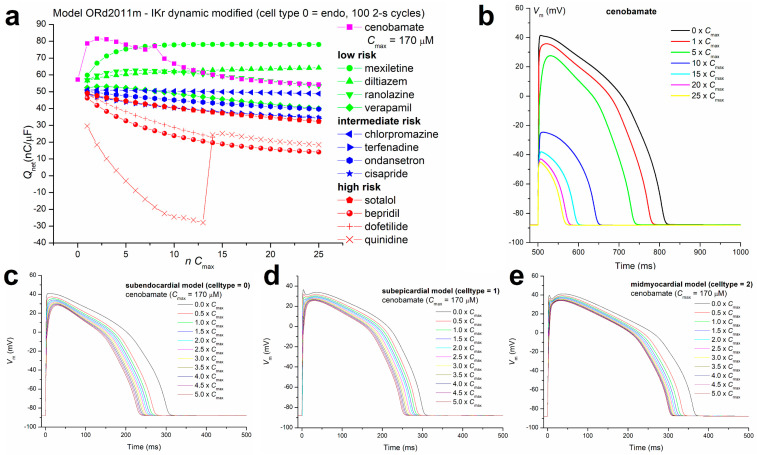
Assessment of proarrhythmogenic risk of cenobamate. (**a**) *Q*_net_ for cenobamate and a 12-compound CiPA training set described in Dutta et al., 2017 [18], computed by simulations with a modified O’Hara–Rudy 2011 model including pharmacology data, as described in Section S2 of Supplementary Materials; (**b**) AP shape generated by the model for different cenobamate concentrations in the range of 0–25 × *C*_max_ (*C*_max_ = maximal effective therapeutic plasma concentration); (**c**–**e**) AP computed with the same model for the three cardiomyocyte types defined in the O’Hara–Rudy 2011 model (subendocardial, subepicardial, and midmyocardial) in a more realistic range of cenobamate concentrations (0–5 × *C*_max_).

With this edited script, we computed *Q*_net_ values for cenobamate concentrations ranging between 1 and 25 × *C*_max_ (170 µM). These values are plotted in Figure 6a, along with those for a 12-compound drug panel used as the Comprehensive in vitro Proarrhythmia Assay (CiPA) [10,20] training set [21]. These large *Q*_net_ values, which are the result of the predominant inhibition by cenobamate of depolarizing ion currents (*I*_Na_ and *I*_CaL_) and negligible effects on repolarizing currents (*I*_Kr_ and *I*_Ks_), demonstrate convincingly the lack of torsadogenic risk for cenobamate. The action potential (AP) shapes generated by the script, using the default parameter set for subendocardial ventricular cardiomyocytes (cell type = 0) at different cenobamate concentrations and in control conditions, are illustrated in Figure 6b. At 1 × *C*_max_, a clinically relevant concentration, there is already a significant shortening of AP duration (computed APD90 of 270.5 ms vs. 304.0 ms in control conditions), consistent with the QT_c_ shortening recorded in experiments on animal models and clinical trials and with our experimental data on APD90 shortening by cenobamate 200 µM in hiPSC-CM. In Figure 6c,d,e, we illustrate the effects of cenobamate concentrations in the range 1–5 × *C*_max_ on AP shape and duration for the three cardiomyocyte variants of the O’Hara–Rudy 2011 model: subendocardial (cell type = 0), subepicardial (cell type = 1), and midmyocardial (cell type = 2). Interestingly, for the midmyocardial type, deemed to form the major part of ventricular myocardium, APD is shortened but remains above 300 ms, which is similar to experimental findings on QT_c_ interval shortening during cenobamate obtained in clinical study YKP3089C020 performed on healthy volunteers ([9], p. 11).

### 3.4. Computational Study of Cenobamate Effects on AP Propagation in Ventricular Tissue

Although cenobamate does not present any significant torsadogenic risk, its local anesthetic-like properties and relatively strong inhibition of the human cardiac Na^+^ channel isoform hNav1.5 imply that it may alter the rapid depolarization (phase 0) of the AP, particularly in ventricular cardiomyocytes, subsequently slowing down conduction velocity and possibly creating favorable conditions for the activation of reentry circuits in pathological myocardium. To explore in more detail these potentially hazardous effects, we performed some theoretical computations to assess conditions prone to triggering ventricular reentry arrhythmias (Section S3 of Supplementary Materials) as well as simulations with a 1D model of the human ventricular myocardium using the modified ventricular cardiomyocyte electrophysiology model described above. These preliminary computations prove that it is conceivable to have, in the pathological myocardium, short (less than 4.5 cm) potential (latent) reentry circuits resulting from the combination of a fast (normal) and slow (pathologic) pathway, united at the ends, which would not give reentry in basal conditions but would lead to reentry in the presence of cenobamate. For example, at a cenobamate concentration of 200 µM, the minimum length of a two-pathway circuit that would allow slow-to-fast reentry will decrease to only 6 mm for an estimated refractory period on the fast pathway of 300 ms.

**Figure 7:**
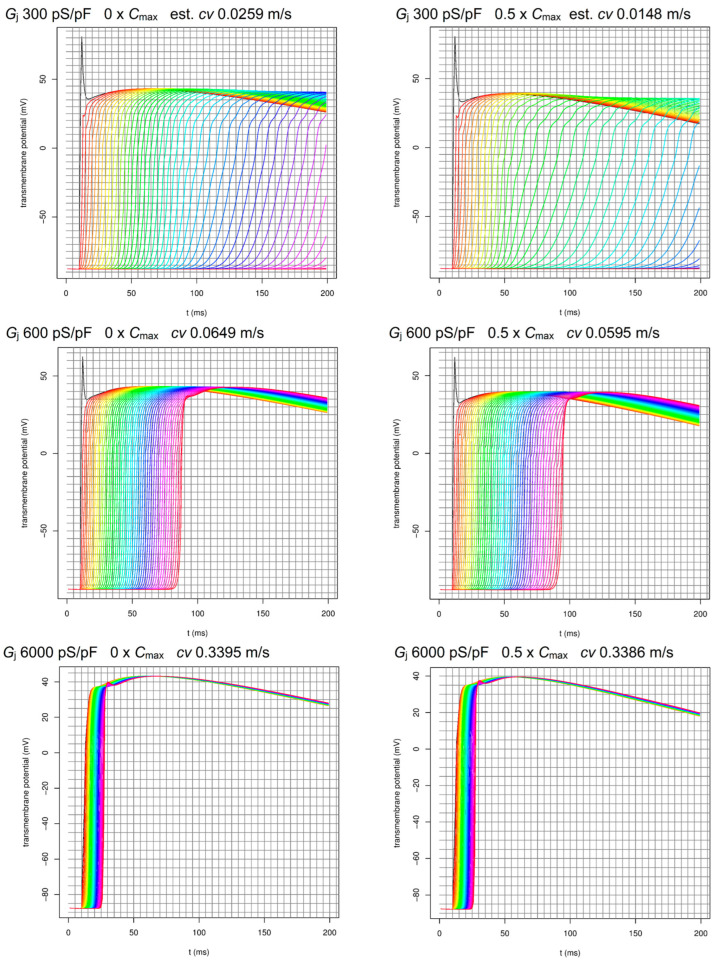
AP propagation along a linear string of 50 ventricular cardiomyocytes simulated with a modified O’Hara–Rudy 2011 model with included pharmacology data, in different conditions (*G*_j_—gap junction conductance; *C*_max_—maximal effective therapeutic plasma concentration of cenobamate = 170 µM; *cv*—conduction velocity).

For the 1D ventricular tissue propagation model, we simulated a linear string of 50 cardiomyocytes with the default geometry parameters of the O’Hara–Rudy 2011 model united end-to-end by intercalated discs with gap junction conductances *G*_j_ varying between highly altered pathological values (lower limit 300 pS/pF) and normal physiological values (upper limit 6000 pS/pF). The modified cell electrophysiology O’Hara–Rudy model with included cenobamate pharmacology data developed by us was used for each cell in the string, and the total current density equation was supplemented with a term describing longitudinal intermyocyte current flow obtained by multiplying *G*_j_ with the actual potential difference between adjacent cardiomyocytes. Simulation results with different parameter sets (*G*_j_ between 300 and 6000 pS/pF and cenobamate concentration between 0 and 6 × *C*_max_) are presented in Tables S9 and S10 of the Supplementary Materials, and for some selected relevant values in Figure 7.

## 4. Discussion

By using a combination of experimental in vitro assays, theoretical computations, and computer simulations, we succeeded in evidencing some novel pharmacological properties of cenobamate and to explore their potential arrhythmogenic consequences, particularly in the pathological myocardium with altered gap junction connectivity and slow conduction velocity. The specific and relatively strong inhibitory effects of the drug on cardiac voltage-dependent Na^+^ channels (hNav1.5) and L-type Ca^2+^ channels were a bit surprising, given that previous studies performed either on neuronal TTX-sensitive hNav1.7 channels stably expressed in HEK293 cells or on newborn rat CA3 hippocampal neurons found a relatively weak inhibitory potency on the peak (transient) Na^+^ current (estimated IC50 > 500 µM), and a much stronger effect on the late (persistent) *I*_Na_, with IC50 values of 41.8 µM ([5] p. 8) and 53.1 µM [22], respectively. This strong inhibition by cenobamate of persistent *I*_Na_ is similar to that exerted by other antiseizure drugs: IC50 2.0 µM for riluzole, 79.2 µM for carbamazepine, and 118.8 µM for lamotrigine ([5] p. 8). This may explain the peculiar effectiveness of this medication in genetic epilepsies with gain-of-function mutations in the SCN1A gene and increased persistent *I*_Na_ [23]. In our experiments on hNav1.5 in the presence of anemone sea toxin ATX-II, we also noticed the strong inhibition by cenobamate of the persistent Na^+^ current, with a similar apparent IC50 of 46.5 µM. Such effects on multiple human cardiac ion channels could represent an argument for the adequacy of the mechanistic in vitro approach of the CiPA paradigm [10,20,21], which is currently only an optional method under the recently adopted S7B/E14 Q&A cardiac safety testing guidelines, and is still often limited to in vitro hERG screening [24].

Given the significant inhibition of the peak Nav1.5 *I*_Na_ current, with an estimated apparent IC_50_ below 100 µM, which is in a clinically relevant range of concentrations, we considered it important to perform an in-depth study of hNav1.5 channel block by cenobamate, including state-dependent blocking kinetics and affinities, and other relevant effects. Indeed, we noticed significant shifts in the voltage dependence of both the activation and steady-state inactivation of *I*_Na_ in the presence of cenobamate at 200 µM. One of the most important published studies on the effects of cenobamate on neuronal *I*_Na_, that of Nakamura et al., 2019 [22], found a similar hyperpolarizing shift of the voltage dependence of inactivation, with a −6 mV change in half-inactivation potential at 100 µM cenobamate, with no significant change in the slope factor and voltage dependence of activation. The authors suggested that the main inhibitory effect of cenobamate on Na^+^ channels may be due to the modulation of the fast voltage-dependent inactivation. Via an elegant method, they also estimated a high affinity for the inactivated conformation of Na^+^ channels (*K*_I_ = 48.2 µM) and a low affinity for the resting (closed) conformation (*K*_R_ = 797.8 µM). This state dependence of drug binding to Na^+^ channels also led us to assume, in the case of hNav1.5, different blocker affinities and hence blocking–unblocking rates for the three main conformations: open (O), inactivated (I), and closed (C); this hypothesis was adopted in previous state-dependent blocking kinetics studies on Nav1.5, particularly those for the class Ib antiarrhythmic lidocaine [17,25]. Two main mechanistic hypotheses have been proposed for Na^+^ channel block and the use-dependent inhibition of peak Na^+^ current by local anesthetics [26]: the modulated receptor hypothesis (MRH) [15,16] and the guarded receptor hypothesis (GRH) [27], as discussed in O’Leary ME and Chahine M, 2017 [28]. The MRH postulates different drug binding affinities for different conformations or states of the channels: high for the open (O) conformation, even higher for the inactivated (I) conformation, and a very low affinity for the closed (C) conformation; drug binding does not affect the gating kinetics itself. The GRH supposes a fast aqueous pathway to the blocking site accessible for charged compounds in the open state only, the block being thus modulated by both the activation and inactivation gates. Given that cenobamate is a neutral molecule at physiological pH (physiological charge 0; strongest acidic pKa 14.28; strongest basic pKa −1.7; logP 1.66; water solubility 0.936 mg/dL—https://go.drugbank.com/drugs/DB06119, accessed on 10 October 2024), we postulated that the blocking site on hNav1.5 channels should also be accessible via a lateral lipophilic pathway and binding/unbinding should also occur in the inactivated and closed conformations; therefore, we considered the MRH model as the most adequate in this case. Mutagenesis studies have shown that the voltage sensors (transmembrane helix S4) of Na^+^ channel domains I–III contribute the activation gating current [29,30,31,32], while S4 of domain IV is largely involved in fast voltage-dependent inactivation [31,33]. In addition, it has been shown that some highly conserved aromatic residues located on S6 of domain IV, a Phe near the inner end of the selectivity filter [34,35] and a Tyr near the inner activation gate [36], are the main determinants of local anesthetic binding [37]. Therefore, our supplementary working hypothesis in resolving the state-dependent Nav1.5-cenobamate binding affinity by assuming equal ratios of open vs. inactivated-specific dissociation constant for cenobamate and lidocaine, although weak, has some logical grounds. Of course, it will be very important for further studies to more accurately assess this state-dependent affinity and blocking kinetics, if possible via model-independent approaches and avoiding simultaneous multi-parameter optimization methods, since the real mechanism of inhibition is still putative and incompletely validated. Some reports claimed that local anesthetics, like lidocaine and its charged derivatives, also reduce the *I*_Na_ gating current and charge, postulating that the main inhibitory effect occurs via gating charge immobilization [38,39].

Another difficult problem is related to the effects of temperature on Nav1.5 inhibition by cenobamate. Due to the small volume of cell suspension used in our experiments and lack of continuous perfusion, we could not perform recordings at physiological temperature (PT: 35–37 °C). There are also only a few published studies describing the pharmacological effects on Nav1.5 channels at PT. Our own and other studies proved an approximately four-fold reduction in the apparent IC_50_ of chloroquine binding on Nav1.5, and more than a two-fold reduction for hydroxychloroquine when comparing experimental results on peak Na^+^ current at PT vs. room temperature, i.e., a stronger binding at higher temperature [11,40], and even higher differences for the effects of these two drugs on late Na^+^ currents [40]. Another study exploring the inhibition of different Nav isoforms by cannabidiol showed decreased affinity and higher IC50 values at higher temperatures for Nav1.2 [41], but in this case multiple binding sites were present, as indicated by the Hill coefficients of ~3, some of them located at the fenestration between domains I and IV or close to the pocket for the inactivation particle [42]. By similarity with temperature effects on the blocking and unblocking rates of hERG channel pore blockers [43], we can assume that Nav1.5 central cavity blockers increase both their binding and unbinding rates with increasing temperature. On the other hand, Nav channel gating is also temperature-dependent. A recent study on multiple Nav isoforms evidenced the hyperpolarizing shifts of the voltage dependence of activation with increasing temperature, amounting up to −10 mV for hNav1.5 upon changing from 15 °C to 35 °C [44]. Our own unpublished data, including voltage-clamp recordings on hNav1.5 at 22 °C and 35 °C, resulted in estimates of the Nav1.5 activation gating temperature coefficient *Q*_10_ of 1.98 and the inactivation *Q*_10_ of 1.2; measuring the area under the curve for the Nav1.5 current at these temperatures yielded a ratio of 0.64 (at physiological vs. room temperature), i.e., the activation, but also the inactivation, kinetics are faster. For charged compounds that can only access the central cavity blocking site during the open state, this would imply a lower binding at higher temperatures, whereas for neutral compounds that can also access this site via the lipophilic lateral pathway, binding would be stronger if the affinity for the inactivated conformation is higher compared to that for the open conformation. We may add to these complex effects the temperature dependence of drug protonation/unprotonation, if the charged and neutral forms feature different binding affinities, as in the case of lidocaine [45]. Due to the multiplicity of mechanisms and opposed direction of effects, for local anesthetic-type Nav blockers it is quasi-impossible to extrapolate experimental results on drug inhibition at room temperature to predict changes in affinity at PT [46].

Turning to the cardiac consequences of the inhibitory effects of cenobamate on multiple ion channels that we identified in this study, we were of course pleased to retrieve, in single-cardiomyocyte electrophysiology simulations, an AP shortening similar to the QT interval shortening greater than 20 ms found in 31% of healthy volunteers who received a 200 mg oral dose of cenobamate and 66% of those who received 500 mg vs. 6–17% in negative control subjects given a placebo in study YKP3089C020 ([9] p. 11). In view of our data, this cardiac depolarization shortening is a combined result of the inhibition of persistent *I*_Na_ and *I*_CaL_, both directly and indirectly, by the subsequent reduction in myocyte sarcoplasmic calcium concentration and current via Na^+^/Ca^2+^ exchangers (NCX), which provide, via the electrogenic exchange of one extruded Ca^2+^ ion for three internalized Na^+^ ions, a net inward current during the AP plateau phase [47].

With high positive *Q*_net_ values that preclude any proarrhythmogenic risk via the inhibition of delayed rectifier K^+^ currents and place the compound in the low torsadogenic risk group (Figure 6a), there is still a possibility that cenobamate will trigger reentry arrhythmias via inhibitory effects on *I*_Na_ and, subsequently, on conduction velocity, combined with a reduction in AP duration and hence in refractory period. Both preliminary theoretical computations and more elaborate computer simulations on a 1D string of ventricular cardiomyocytes showed that, in the pathological myocardium with more than ten-fold increased intermyocyte gap junction resistance, *I*_Na_ inhibition by cenobamate should exert a marked slowing down of conduction velocity, leading to a significant difference in velocity compared to an adjacent region of the normal myocardium and the possibility of activation of a reentry circuit either by slow-to-fast pathway conduction or by fast-to-slow conduction if AP propagation failed on the slow pathway or the cardiomyocytes are within a narrow vulnerability window for reentry during the late stage of phase 3 [48]. We identified some upper and lower bounds for space and time intervals, conduction velocity differences, and pacing frequencies that would result in reentry, but of course this exploration has to be continued using more sophisticated and realistic 2D or 3D myocardium models, given the extremely large variability in velocity and refractoriness induced by conditions such as myocardial ischemia [49]. Early simulation studies of cardiac AP propagation identified a minimal 5- to 10-fold gap junction resistance increase as a necessary condition for unidirectional block ([50], cited by [51]), but these conditions can be easily met, particularly during ischemia-related acidosis [52,53,54,55]. We also note that 1D cardiomyocyte tissue models at different levels of complexity have been successfully used to study various problems, including unidirectional block [25,56,57,58,59,60].

Finally, it is worth mentioning that the complex pharmacokinetics and biodisponibility of cenobamate play an important role for the in vivo and clinical relevance of these effects. Complex studies were performed to characterize the single-dose pharmacokinetics and interactions with bupropion, midazolam, warfarin, and omeprazole [61] or to assess the mass balance and metabolic profile using a radioactively labeled compound [62]. Accurate and validated liquid chromatography–mass spectrometry (UHPLC-MS/MS) protocols were developed, using, for example, a water–acetonitrile multistep elution gradient for cenobamate extraction from plasma samples [63]. Distribution and bioavailability assays found different species-specific percentages of protein binding of cenobamate, ranging from 35.4% in rabbit and 43.2% in mouse to 61.0% in human and 60.7–70.7% in non-human primates ([5] p.10). Given the average plasma protein binding of 60%, the free compound levels may be lower than the total concentrations assessed during pharmacokinetic studies, even at maximal effective plasma therapeutic concentrations; therefore, the biological effects may be limited and the risks reduced. In assessing clinical effects, a 0.5 × *C*_max_ concentration may provide the most relevant results.

## 5. Conclusions

Cenobamate, applied at concentrations in the clinically relevant range, resulted in the significant inhibition of Nav1.5 and Cav1.2 channels, which may explain the dose-dependent QT_c_ shortening effects and the interdiction for administering the drug to patients with short QT syndrome.

These blocking effects, particularly on Nav1.5, although generally deemed to be antiarrhythmic, may also trigger arrhythmias in pathological conditions such as myocardial ischemia or other structural heart diseases by slowing down conduction and favoring reentry [64].

## Figures and Tables

**Table 1:** Estimated *τ_ib_* obtained by analyzing 4 experiments on hNav1.5-expressing HEK293 cells by applying a voltage protocol for studying the frequency dependence of use-dependent block.

Exp.	*τ_i_* (ms)	*τ_r_* (ms)	*τ_i_*_+*ob*_ (ms)	est. *τ_ib_* (ms)
23o10	2.36	3.31	2.23	4.08
a3o10	2.62	3.76	3.04	3.83
b3o10	2.93	5.13	2.94	4.94
c3o10	1.95	3.04	1.73	3.25
**Mean ± SD**	**2.47 ± 0.41**	**3.81 ± 0.93**	**2.49 ± 0.62**	**4.03 ± 0.70**

**Table 2:** Kinetics of cenobamate block/unblock of the open conformation (O) of hNav1.5 channels estimated by analysis of recordings with the Nav standard voltage protocol. The upper part of the table shows apparent inactivation time constants (*τ*_inact_) measured in the same cell initially and at 3 min after application of cenobamate dissolved in vehicle solution, and the lower part apparent *τ*_inact_ measured initially and at 3 min after application of the same amount of vehicle solution only (negative control experiments).

**Exp. No.**	***τ*_inact_ Initial (ms)**	***τ*_inact_ Cenobamate (ms)**
23314	1.19	0.67
23317	1.63	0.83
23321 *	3.49	1.21
a3321	0.87	0.56
23324	0.67	0.58
a3324	0.98	0.56
**Mean**	**1.07**	**0.64**
**SD**	**0.36**	**0.12**
**Rate (1/*τ*)**	**0.94**	**1.56**
**Sum of Blocking and Unblocking Rates from Open Conformation**		
**(*k*_ob_ + *k*_ob_^−1^) (ms^−1^):**		**1.56 − 0.94 = 0.62 ms^−1^**
**Exp. No.**	***τ*_inact_ Initial (ms)**	***τ*_inact_ Control (ms)**
23311	1.96	1.86
23325	1.89	1.88
23517	4.00	4.06
23519	2.09	2.04
a3519	0.78	0.79
**Mean**	**2.15**	**2.12**
**SD**	**1.16**	**1.19**

* values not included in further analysis due to artifacts given by adjacent cell connected by gap junctions.

## Data Availability

Except for the Supplementary Materials, the raw experimental and simulation data are available from the corresponding author upon reasonable request.

## Supplementary Materials

The following supporting information can be downloaded at https://www.mdpi.com/article/10.3390/biom14121582/s1, Figure S1. Voltage independence of the inhibitory effect of cenobamate 200 µM on *I*_Na_ peak amplitude; Figure S2. Peak and late *I*_Na_ elicited with a CiPA-like voltage protocol; Figure S3. Voltage protocol Nav_freq_dep.pro for assessment of use-dependent block; Table S1. Summary of experimental results concerning the effects of cenobamate 200 µM and vehicle DMSO solution in control experiments on peak *I*_Na_ amplitude; Table S2. Summary of experimental results concerning the effects of cenobamate at multiple concentrations on peak *I*_Na_ amplitude; Table S3. Summary of experimental results concerning the effects of cenobamate at multiple concentrations on late *I*_Na_; Table S4. Summary of experimental results concerning the effects of cenobamate 200 µM and vehicle DMSO solution in control experiments on peak *I*_CaL_ amplitude; Table S5. Summary of experimental results concerning the effects of cenobamate at multiple concentrations on peak *I*_CaL_ amplitude; Table S6. Summary of experimental results concerning the effects of cenobamate 200 µM and vehicle DMSO solution in control experiments on plateau *I*_Ks_ amplitude; Table S7. Summary of experimental results concerning the effects of cenobamate 200 µM on peak *I*_Kr_ amplitude; Table S8. Summary of experimental results concerning the effects of cenobamate 200 µM on AP parameters in amphotericin-perforated whole-cell patch-clamp experiments on Ncyte^®^ vCardiomyocytes hiPSC-CM; Table S9. Number of cells featuring a propagated AP in computations with a linear string of 50 ventricular cardiomyocytes simulated with a modified O’Hara–Rudy 2011 model with included pharmacology data; Table S10. End-to-end AP propagation time in computations with a linear string of 50 ventricular cardiomyocytes simulated with a modified O’Hara–Rudy 2011 model with included pharmacology data. Section S1. Analysis of variation in fraction of open channels during repeated depolarizing stimuli protocols used to assess use-dependent block; Section S2. Modification of the O’Hara–Rudy 2011 model adapted for pharmacology simulations to include inhibition and data state-dependent blocking/unblocking of Na^+^ channels by cenobamate; Section S3. Preliminary estimate of cenobamate effect on ventricular conduction velocity and the risk of induction of reentry arrhythmia in pathological myocardium with large conduction velocity heterogeneity. References cited in Supplementary Materials [11,14,48,49,50,51].

## References

[1] Rissardo J.P., Caprara A.L.F. (2023). Cenobamate (YKP3089) and drug-resistant epilepsy: A review of the literature. Medicina.

[2] Wheless J.W. (2020). Adjunctive cenobamate for the treatment of focal onset seizures in adults with epilepsy: A critical review. Expert Rev. Neurother..

[3] Löscher W. (2021). Single-target versus multi-target drugs versus combinations of drugs with multiple targets: Preclinical and clinical evidence for the treatment or prevention of epilepsy. Front. Pharmacol..

[4] Sharma R., Nakamura M., Neupane C., Jeon B.H., Shin H., Melnick S.M., Glenn K.J., Jang I.S., Park J.B. (2020). Positive allosteric modulation of GABA(A) receptors by a novel antiepileptic drug cenobamate. Eur. J. Pharmacol..

[5] US Food and Drug Administration (2019). Non-Clinical Review(s). https://www.accessdata.fda.gov/drugsatfda_docs/nda/2019/212839Orig1s000PharmR.pdf.

[6] Roberti R., De Caro C., Iannone L.F., Zaccara G., Lattanzi S., Russo E. (2021). Pharmacology of cenobamate: Mechanism of action, pharmacokinetics, drug-drug interactions and tolerability. CNS Drugs.

[7] Sankar R. (2023). Treatment of status epilepticus: Physiology, pharmacology, and future directions. Epilepsia Open.

[8] Specchio N., Pietrafusa N., Vigevano F. (2021). Is cenobamate the breakthrough we have been wishing for?. Int. J. Mol. Sci..

[9] US Food and Drug Administration (2019). Risk Assessment and Risk Mitigation Review(s). https://www.accessdata.fda.gov/drugsatfda_docs/nda/2019/212839Orig1s000RiskR.pdf.

[10] Sager P.T., Gintant G., Turner J.R., Pettit S., Stockbridge N. (2014). Rechanneling the cardiac proarrhythmia safety paradigm: A meeting report from the Cardiac Safety Research Consortium. Am. Heart J..

[11] Thomet U., Amuzescu B., Knott T., Mann S.A., Mubagwa K., Radu B.M. (2021). Assessment of proarrhythmogenic risk for chloroquine and hydroxychloroquine using the CiPA concept. Eur. J. Pharmacol..

[12] Mann S.A., Heide J., Knott T., Airini R., Epureanu F.B., Deftu A.-F., Deftu A.-T., Radu B.M., Amuzescu B. (2019). Recording of multiple ion current components and action potentials in human induced pluripotent stem cell-derived cardiomyocytes via automated patch-clamp. J. Pharmacol. Toxicol. Methods.

[13] Scheel O., Frech S., Amuzescu B., Eisfeld J., Lin K.H., Knott T. (2014). Action potential characterization of human induced pluripotent stem cell-derived cardiomyocytes using automated patch-clamp technology. Assay Drug Dev. Technol..

[14] O’Hara T., Virág L., Varró A., Rudy Y. (2011). Simulation of the undiseased human cardiac ventricular action potential: Model formulation and experimental validation. PLoS Comput. Biol..

[15] Courtney K.R. (1975). Mechanism of frequency-dependent inhibition of sodium currents in frog myelinated nerve by the lidocaine derivative GEA. J. Pharmacol. Exp. Ther..

[16] Hille B. (1977). Local anesthetics: Hydrophilic and hydrophobic pathways for the drug-receptor reaction. J. Gen. Physiol..

[17] Cardona K., Trénor B., Moltó G., Martínez M., Ferrero J.M., Starmer F., Saiz J. (2010). Exploring the role of pH in modulating the effects of lidocaine in virtual ischemic tissue. Am. J. Physiol. Heart Circ. Physiol..

[18] Dutta S., Chang K.C., Beattie K.A., Sheng J., Tran P.N., Wu W.W., Wu M., Strauss D.G., Colatsky T., Li Z. (2017). Optimization of an in silico cardiac cell model for proarrhythmia risk assessment. Front. Physiol..

[19] Li Z., Dutta S., Sheng J., Tran P.N., Wu W., Chang K., Mdluli T., Strauss D.G., Colatsky T. (2017). Improving the in silico assessment of proarrhythmia risk by combining hERG (human ether-a-go-go-related gene) channel-drug binding kinetics and multichannel pharmacology. Circ. Arrhythmia Electrophysiol..

[20] Fermini B., Hancox J.C., Abi-Gerges N., Bridgland-Taylor M., Chaudhary K.W., Colatsky T., Correll K., Crumb W., Damiano B., Erdemli G. (2016). A new perspective in the field of cardiac safety testing through the comprehensive in vitro proarrhythmia assay paradigm. J. Biomol. Screen..

[21] Colatsky T., Fermini B., Gintant G., Pierson J.B., Sager P., Sekino Y., Strauss D.G., Stockbridge N. (2016). The comprehensive in vitro proarrhythmia assay (CiPA) initiative—Update on progress. J. Pharmacol. Toxicol. Methods.

[22] Nakamura M., Cho J.H., Shin H., Jang I.S. (2019). Effects of cenobamate (YKP3089), a newly developed anti-epileptic drug, on voltage-gated sodium channels in rat hippocampal CA3 neurons. Eur. J. Pharmacol..

[23] Stafstrom C.E. (2007). Persistent sodium current and its role in epilepsy. Epilepsy Curr..

[24] Darpo B., Leishman D.J. (2023). The new S7B/E14 Q&A document provides additional opportunities to replace the thorough QT study. J. Clin. Pharmacol..

[25] Moreno J.D., Zhu Z.I., Yang P.C., Bankston J.R., Jeng M.T., Kang C., Wang L., Bayer J.D., Christini D.J., Trayanova N.A. (2011). A computational model to predict the effects of class I anti-arrhythmic drugs on ventricular rhythms. Sci. Transl. Med..

[26] Wang K.G., Stricharts G.R. (2012). State-dependent inhibition of sodium channels by local anesthetics: A 40-year evolution. Biochem. (Mosc.) Suppl. Ser. A Membr. Cell Biol..

[27] Starmer C.F., Grant A.O., Strauss H.C. (1984). Mechanisms of use-dependent block of sodium channels in excitable membranes by local anesthetics. Biophys. J..

[28] O’Leary M.E., Chahine M., Chahine M. (2017). Mechanisms of drug binding to voltage-gated sodium channels. Voltage-Gated Sodium Channels: Structure, Function and Channelopathies.

[29] Capes D.L., Goldschen-Ohm M.P., Arcisio-Miranda M., Bezanilla F., Chanda B. (2013). Domain IV voltage-sensor movement is both sufficient and rate limiting for fast inactivation in sodium channels. J. Gen. Physiol..

[30] Chanda B., Bezanilla F. (2002). Tracking voltage-dependent conformational changes in skeletal muscle sodium channel during activation. J. Gen. Physiol..

[31] Chen L.Q., Santarelli V., Horn R., Kallen R.G. (1996). A unique role for the S4 segment of domain 4 in the inactivation of sodium channels. J. Gen. Physiol..

[32] Sheets M.F., Kyle J.W., Kallen R.G., Hanck D.A. (1999). The Na channel voltage sensor associated with inactivation is localized to the external charged residues of domain IV, S4. Biophys. J..

[33] Chahine M., George A.L., Zhou M., Ji S., Sun W., Barchi R.L., Horn R. (1994). Sodium channel mutations in paramyotonia congenita uncouple inactivation from activation. Neuron.

[34] Ahern C.A., Eastwood A.L., Dougherty D.A., Horn R. (2008). Electrostatic contributions of aromatic residues in the local anesthetic receptor of voltage-gated sodium channels. Circ. Res..

[35] Pless S.A., Galpin J.D., Frankel A., Ahern C.A. (2011). Molecular basis for class Ib anti-arrhythmic inhibition of cardiac sodium channels. Nat. Commun..

[36] Ragsdale D.S., McPhee J.C., Scheuer T., Catterall W.A. (1994). Molecular determinants of state-dependent block of Na+ channels by local anesthetics. Science.

[37] Lipkind G.M., Fozzard H.A. (2005). Molecular modeling of local anesthetic drug binding by voltage-gated sodium channels. Mol. Pharmacol..

[38] Hanck D.A., Makielski J.C., Sheets M.F. (2000). Lidocaine alters activation gating of cardiac Na channels. Pflügers Arch. Eur. J. Physiol..

[39] Sheets M.F., Fozzard H.A., Lipkind G.M., Hanck D.A. (2010). Sodium channel molecular conformations and antiarrhythmic drug affinity. Trends Cardiovasc. Med..

[40] Delaunois A., Abernathy M., Anderson W.D., Beattie K.A., Chaudhary K.W., Coulot J., Gryshkova V., Hebeisen S., Holbrook M., Kramer J. (2021). Applying the CiPA Approach to Evaluate Cardiac Proarrhythmia Risk of some Antimalarials Used Off-label in the First Wave of COVID-19. Clin. Transl. Sci..

[41] Ghovanloo M.R., Shuart N.G., Mezeyova J., Dean R.A., Ruben P.C., Goodchild S.J. (2018). Inhibitory effects of cannabidiol on voltage-dependent sodium currents. J. Biol. Chem..

[42] Li Z., Wu Q., Yan N. (2024). A structural atlas of druggable sites on Na(v) channels. Channels.

[43] Windley M.J., Lee W., Vandenberg J.I., Hill A.P. (2018). The Temperature Dependence of Kinetics Associated with Drug Block of hERG Channels Is Compound-Specific and an Important Factor for Proarrhythmic Risk Prediction. Mol. Pharmacol..

[44] Kriegeskorte S., Bott R., Hampl M., Korngreen A., Hausmann R., Lampert A. (2023). Cold and warmth intensify pain-linked sodium channel gating effects and persistent currents. J. Gen. Physiol..

[45] Schwarz W., Palade P.T., Hille B. (1977). Local anesthetics. Effect of pH on use-dependent block of sodium channels in frog muscle. Biophys. J..

[46] Johns J.A., Anno T., Bennett P.B., Snyders D.J., Hondeghem L.M. (1989). Temperature and voltage dependence of sodium channel blocking and unblocking by O-demethyl encainide in isolated guinea pig myocytes. J. Cardiovasc. Pharmacol..

[47] Noble D. (2007). From the Hodgkin-Huxley axon to the virtual heart. J. Physiol..

[48] Rudy Y. (1995). Reentry: Insights from theoretical simulations in a fixed pathway. J. Cardiovasc. Electrophysiol..

[49] Kléber A.G., Rudy Y. (2004). Basic mechanisms of cardiac impulse propagation and associated arrhythmias. Physiol. Rev..

[50] Rudy Y., Quan W.L. (1987). A model study of the effects of the discrete cellular structure on electrical propagation in cardiac tissue. Circ. Res..

[51] Cooklin M., Wallis W.R., Sheridan D.J., Fry C.H. (1998). Conduction velocity and gap junction resistance in hypertrophied, hypoxic guinea-pig left ventricular myocardium. Exp. Physiol..

[52] Bukauskas F.F., Peracchia C. (1997). Two distinct gating mechanisms in gap junction channels: CO_2_-sensitive and voltage-sensitive. Biophys. J..

[53] Carmeliet E. (1999). Cardiac ionic currents and acute ischemia: From channels to arrhythmias. Physiol. Rev..

[54] De Groot J.R., Coronel R. (2004). Acute ischemia-induced gap junctional uncoupling and arrhythmogenesis. Cardiovasc. Res..

[55] Noma A., Tsuboi N. (1987). Dependence of junctional conductance on proton, calcium and magnesium ions in cardiac paired cells of guinea-pig. J. Physiol..

[56] Bondarenko V.E., Rasmusson R.L. (2007). Simulations of propagated mouse ventricular action potentials: Effects of molecular heterogeneity. Am. J. Physiol. Heart Circ. Physiol..

[57] Inada S., Hancox J.C., Zhang H., Boyett M.R. (2009). One-dimensional mathematical model of the atrioventricular node including atrio-nodal, nodal, and nodal-his cells. Biophys. J..

[58] Livshitz L., Rudy Y. (2009). Uniqueness and stability of action potential models during rest, pacing, and conduction using problem-solving environment. Biophys. J..

[59] Shaw R.M., Rudy Y. (1995). The vulnerable window for unidirectional block in cardiac tissue: Characterization and dependence on membrane excitability and intercellular coupling. J. Cardiovasc. Electrophysiol..

[60] Tsumoto K., Ashihara T., Haraguchi R., Nakazawa K., Kurachi Y. (2011). Roles of subcellular Na+ channel distributions in the mechanism of cardiac conduction. Biophys. J..

[61] Greene S.A., Kwak C., Kamin M., Vernillet L., Glenn K.J., Gabriel L., Kim H.W. (2022). Effect of cenobamate on the single-dose pharmacokinetics of multiple cytochrome P450 probes using a cocktail approach in healthy subjects. Clin. Transl. Sci..

[62] Vernillet L., Greene S.A., Kim H.W., Melnick S.M., Glenn K. (2020). Mass balance, metabolism, and excretion of cenobamate, a new antiepileptic drug, after a single oral administration in healthy male subjects. Eur. J. Drug Metab. Pharmacokinet..

[63] Charlier B., Coglianese A., Operto F.F., Coppola G., de Grazia U., Menna P., Filippelli A., Dal Piaz F., Izzo V. (2022). Development and validation of a UHPLC-MS/MS-based method to quantify cenobamate in human plasma samples. Molecules.

[64] Roden D.M. (2011). Pharmacology and toxicology of Nav1.5-class 1 anti-arrhythmic drugs. Card. Electrophysiol. Clin..

