# Supplementary material for: Inhibitory Effects of Cenobamate on Multiple Human Cardiac Ion Channels and Possible Arrhythmogenic Consequences
Source: Biomolecules. 2024 Dec 11;14(12):1582. doi: 10.3390/biom14121582 (PMC11674187; doi:10.3390/biom14121582)
Supplement: Supplementary file 1 [file biomolecules-14-01582-s001.zip › biomolecules-3334178-supplementary.pdf]

Supplementary Materials

Table S1. Summary of experimental results concerning the effects of cenobamate 200 µM and vehicle DMSO solution in control experiments on peak I_{Na} amplitude elicited by applying the standard voltage protocol with depolarizing steps at -10 mV (R_a – access resistance).

**Cenobamate 200 µM**		**Peak Current Measured During step at -10 mV**			
**Exp. No.**	**I_{Na} Peak ini (pA)**	**I_{Na} Peak Drug (pA)**	**% I_{Na} Inhibition**	**% R_a (int/ini)**	**% R_a (fin/ini)**
23314	-6014.86	-2630.19	56.27	125.63	155.36
23317	-4911.77	-1536.8	68.71	85.57	117.05
23321	-10402.5	-1357.64	86.95	116.29	0.00
a3321	-6674.65	-3012.24	54.87	87.77	73.31
23324	-3954.7	-488.118	87.66	133.20	178.79
a3324	-10417	-3881.63	62.74	87.63	101.86
**Mean**	**-7062.58**	**-2151.10**	**69.53**	**106.02**	**104.40**
**SD**	**2754.64**	**1244.05**	**14.63**	**21.53**	**63.50**
control DMSO 0.1%		peak current measured during step at -10 mV			
23311	-4397.27	-4411.13	-0.32	165.22	
23325	-2097.17	-2027.99	3.30		
23517	-3821.92	-3768.29	1.40	117.84	106.84
23519	-6182.90	-6276.36	-1.51	70.95	
a3519	-10974.53	-10863.72	1.01	120.11	126.05
**Mean**	**-5494.76**	**-5469.50**	**0.78**	**118.53**	**116.45**
**SD**	**3392.93**	**3376.64**	**1.82**	**38.50**	**13.59**
**Student's *t* test for independent samples (two-tailed)**			**$p < 0.0001$ $t = 10.35$ (9 *d.f.*)**		

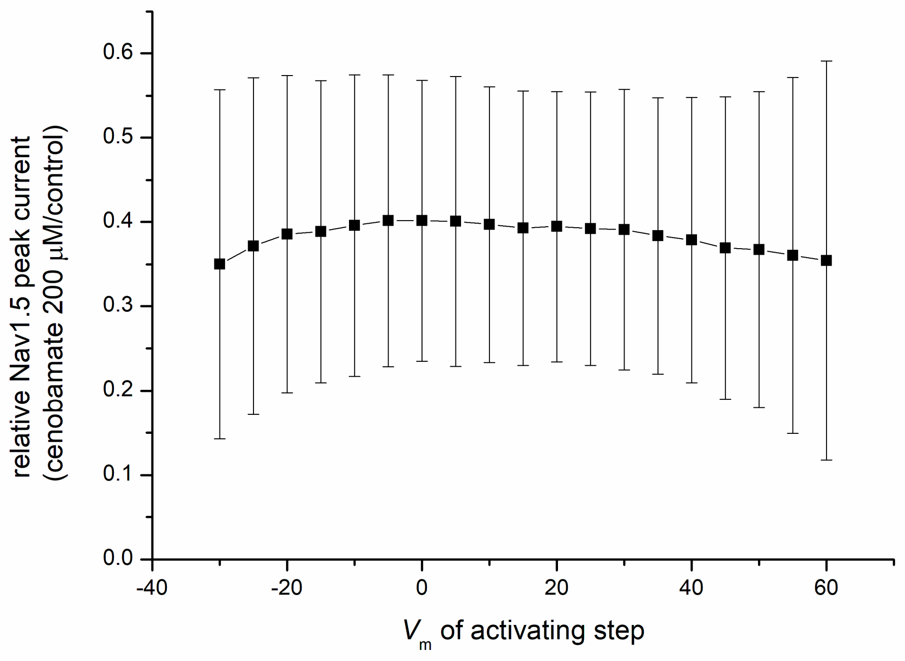

Figure S1. Voltage independence of the inhibitory effect of cenobamate 200 µM on I_{Na} peak amplitude measured at different depolarizing steps (ratio of peak I_{Na} during cenobamate application and initial value at same voltage step in the same experiment, average ± SD of n=6 experiments).

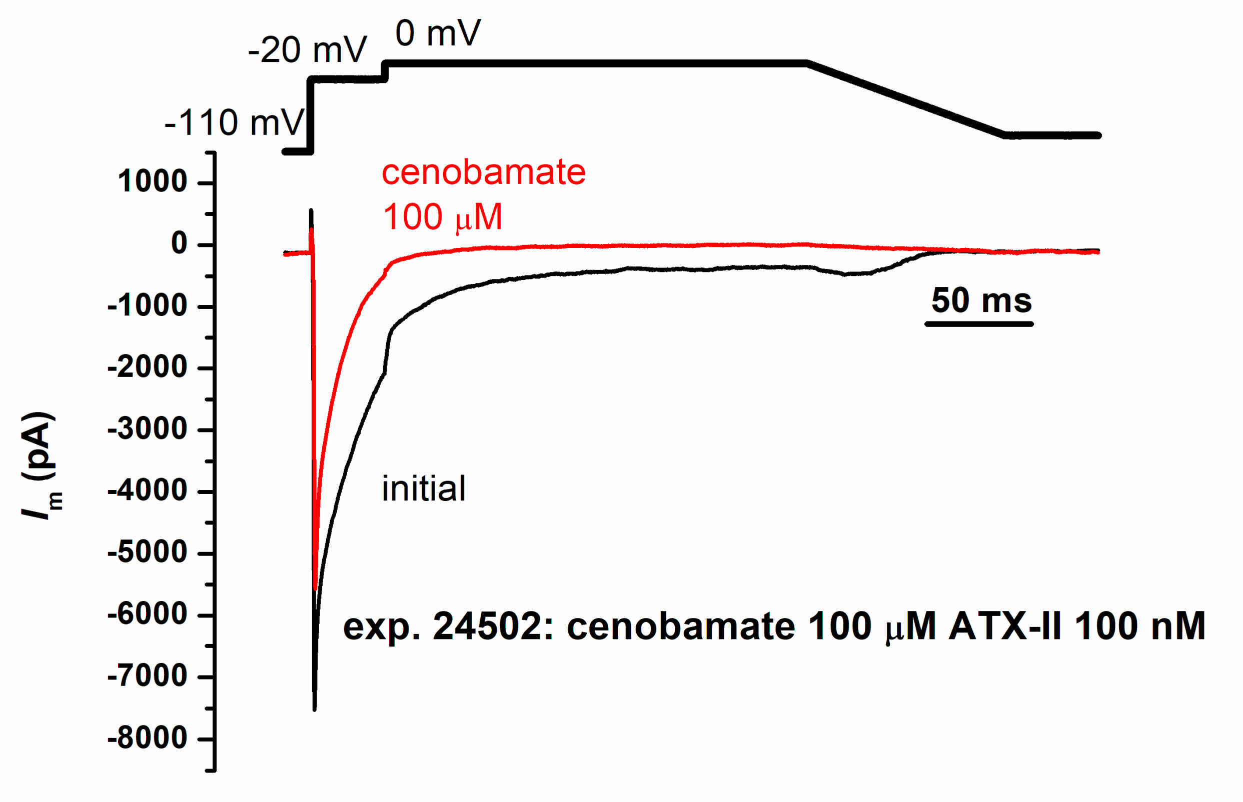

Figure S2. Peak and late I_{Na} elicited with a CiPA-like voltage protocol in a typical experiment with bath solution supplemented with ATX-II 100 nM. Current in control conditions in black, and upon addition of cenobamate to a final concentration of 100 µM in red.

Table S2. Summary of experimental results concerning the effects of cenobamate at multiple concentrations on peak I_{Na} amplitude elicited by applying the standard voltage protocol with depolarizing steps at -10 mV (R_a – access resistance).

Exp. no.	I_{Na} peak ini (pA)	I_{Na} peak drug (pA)	% I_{Na} inhibition	% R_a (fin/ini)
		cenobamate 10 µM		
24612003	-14365.2	-13132.5	8.58	90.56
b4612003	-4930.3	-4447.75	9.79	38.73
24613003	-5596.77	-5035.71	10.02	67.48
b4613003	-6355.59	-5975.95	5.97	121.50
**Mean**	**-5627.55**	**-5153.14**	**8.60**	**75.90**
**SD**	**713.1435**	**770.8376**	**2.27**	**42.02**
		cenobamate 40 µM		
24605003	-4673.31	-2592.01	44.54	56.66
a4605004	-8204.78	-5043.33	38.53	100.26
24606003	-6013.03	-3823.09	36.42	64.79
a4606003	-7665	-5807.5	24.23	67.94
b4606002	-5034.79	-2779.54	44.79	141.24
**Mean**	**-6318.18**	**-4009.09**	**37.70**	**86.18**
**SD**	**1566.763**	**1401.645**	**8.38**	**34.97**
		cenobamate 100 µM		
a4610	-5461.29	-2584.08	52.68	36.38
b4610	-4804.69	-2315.67	51.80	35.06
24611	-2455.04	-1243.29	49.36	62.08
b4611	-9299.01	-5386.66	42.07	72.63
**Mean**	**-5505.01**	**-2882.43**	**48.98**	**51.54**
**SD**	**2839.55**	**1767.12**	**4.81**	**18.77**
		cenobamate 200 µM		

a4524	-4715.88	-1671.96	64.55	33.24
24527	-10215.7	-1290.13	87.37	2.20
a4527	-9294.43	-4215.39	54.65	87.95
24529	-7045.08	-3358.76	52.32	72.55
a4529	-1724	-467.834	72.86	71.73
**Mean**	**-6599.018**	**-2200.81**	**69.86**	**53.53**
**SD**	**3458.92379**	**1542.045**	**13.85**	**35.09**
		cenobamate 1000 µM		
245305	-5449.83	-580.037	89.36	86.24
a45305	-4060.67	-554.657	86.34	51.46
b45306	-5943.81	-617.842	89.61	77.81
246003	-7273.36	-599.365	91.76	80.49
**Mean**	**-5681.92**	**-587.975**	**89.27**	**74.00**
**SD**	**1327.1**	**27.04854**	**2.23**	**15.43**

Table S3. Summary of experimental results concerning the effects of cenobamate at multiple concentrations on late I_{Na} measured at the end of the step at 0 mV using the CiPA-like voltage protocol (R_a – access resistance).

**Exp. no.**	**I_{Na} peak ini (pA)**	**I_{Na} peak drug (pA)**	**% I_{Na} inhibition**	**% R_a (fin/ini)**
		cenobamate 1 µM		
24517005	-24.4387	-22.0434	9.80	177.75
24520002	-174.136	-166.553	4.35	267.69
a4520003	-64.6785	-63.0013	2.59	172.65
**Mean**	**-87.7511**	**-83.8659**	**5.58**	**206.03**
**SD**	**77.46985**	**74.4799**	**3.76**	**53.46**
		cenobamate 10 µM		
24503001	-67.0591	-50.2302	25.10	137.67
24507001	-94.9828	-68.3931	27.99	91.21
a4508001	-38.145	-31.7546	16.75	148.09
b4508001	-70.2307	-47.8626	31.85	137.10
**Mean**	**-67.6044**	**-49.5601**	**25.42**	**128.52**
**SD**	**23.27107**	**15.00054**	**6.41**	**25.38**
		cenobamate 40 µM		
24514001	-144.378	-75.5396	47.68	145.14
a4514002	-70.6974	-40.5042	42.71	151.19
24515001	-215.647	-108.842	49.53	86.99
b4515001	-235.455	-100.181	57.45	134.30
**Mean**	**-166.544**	**-81.2667**	**49.34**	**129.40**
**SD**	**74.91714**	**30.61878**	**6.13**	**29.13**
		cenobamate 100 µM		
24501001	-196.458	-39.0889	80.10	187.24
b4501001	-949.478	-76.1735	91.98	150.22
c4501004	-68.4936	-18.6404	72.79	134.40
24502001	-358.004	-73.643	79.43	105.41
a4502002	-132.643	-35.7356	73.06	265.73
**Mean**	**-341.02**	**-48.66**	**79.47**	**168.60**
**SD**	**356.76**	**25.20**	**7.79**	**61.81**
		cenobamate 200 µM		
b4416001	-147.4	-4.9	96.68	113.47
24423003	-119.7	-6.7	94.40	288.59
a4423001	-42.7	-2.3	94.64	255.50
b4423001	-42.1	-5.5	86.94	124.29
24425002	-65.1	-0.3	99.56	71.02
**Mean**	**-83.4**	**-3.9**	**94.44**	**170.57**
**SD**	**47.7**	**2.6**	**4.68**	**95.46**

Table S4. Summary of experimental results concerning the effects of cenobamate 200 µM and vehicle DMSO solution in control experiments on peak I_{CaL} amplitude elicited by applying the standard voltage protocol with depolarizing steps at 0 mV (R_a – access resistance).

**cenobamate 200 uM**		**peak current measured during step at 0 mV**		
**Exp. no.**	**I_{CaL} peak ini (pA)**	**I_{CaL} peak drug (pA)**	% I_{CaL} inhibition	% R_a (int-fin/ini)
a3831	-378.5	-257.4	31.99	195.90
b3831	-1374.7	-638.1	53.58	113.01
23909	-937.881	-714.989	23.77	201.87
a3909	-1313.97	-687.065	47.71	173.80
**Mean**	**-1001.26**	**-574.39**	**39.26**	**171.14**
**SD**	**457.93**	**213.70**	**13.78**	**40.59**
control DMSO 0.1%		peak current measured during step at 0 mV		
c3831	-272.2	-264.9	2.68	144.26
a3922	-778.102	-774.697	0.44	106.27
b3o13	-40.6075	-38.0993	6.18	455.90
**Mean**	**-363.64**	**-359.23**	**3.10**	**235.48**
**SD**	**377.15**	**377.25**	**2.89**	**191.84**
**Mann-Whitney test, two-tailed**			**$p = 0.0571$**	

Table S5. Summary of experimental results concerning the effects of cenobamate at multiple concentrations on peak I_{CaL} amplitude elicited by applying the standard voltage protocol with depolarizing steps at 0 mV (R_a – access resistance).

**Exp. no.**	**I_{CaL} peak ini (pA)**	**I_{CaL} peak drug (pA)**	**% I_{CaL} inhibition**	**% R_a (fin/ini)**
		cenobamate 20 µM		
a4801002	-440.674	-434.57	1.39	195.08
a4802002	-835.442	-731.862	12.40	310.69
24813002	-395.508	-371.094	6.17	475.11
**Mean**	**-557.21**	**-512.51**	**6.65**	**326.96**
**SD**	**242.01**	**192.60**	**5.52**	**140.73**
		cenobamate 100 µM		
24717002	-2093.730	-1858.350	11.24	94.85
b4717002	-651.089	-576.882	11.40	131.44
24718002	-86.220	-77.097	10.58	208.27
b4718002	-750.505	-660.188	12.03	131.44
**Mean**	**-821.24**	**-730.11**	**11.31**	**141.77**
**SD**	**755.22**	**669.19**	**0.60**	**41.35**
		cenobamate 200 µM		
a3831	-378.500	-257.400	31.99	319.98
23909	-937.881	-714.989	23.77	237.48
a3909	-1314.600	-688.305	47.64	181.11
24703003	-144.653	-88.501	38.82	113.87
a4704002	-744.629	-518.799	30.33	224.84
**Mean**	**-866.69**	**-499.73**	**34.51**	**215.46**
**SD**	**471.07**	**237.26**	**9.09**	**75.83**
		cenobamate 400 µM		
a4722002	-551.880	-336.945	38.95	71.33
b4723002	-868.530	-510.254	41.25	105.90
24724002	-139.160	-73.242	47.37	414.10
24726002	-1208.500	-720.215	40.40	156.47
**Mean**	**-692.02**	**-410.16**	**41.99**	**186.95**
**SD**	**455.78**	**273.88**	**3.71**	**155.42**
		cenobamate 1000 µM		
24726002	-310.059	-70.190	77.36	333.21
24730002	-478.328	-199.014	58.39	204.76

a4730002	-117.247	-27.525	76.52	185.75
24731002	-935.059	-440.674	52.87	187.70
b4718002	-2103.020	-808.441	61.56	187.72
**Mean**	**-788.74**	**-309.17**	**65.34**	**219.83**
**SD**	**794.53**	**322.15**	**11.04**	**63.85**

Table S6. Summary of experimental results concerning the effects of cenobamate 200 µM and vehicle DMSO solution in control experiments on plateau I_{Ks} amplitude measured during a depolarizing step at +60 mV (R_a – access resistance).

**cenobamate 200 µM**		**current measured at end of 3-s pulse to +60 mV**		
**Exp. no.**	**I_{Ks} plateau ini (pA)**	**I_{Ks} plateau drug (pA)**	**% I_{Ks} inhibition**	**% R_a (int-fin/ini)**
b3421	345.5	271	21.56	195.90
a3426	1398.9	878.9	37.17	106.04
b3426	3302	2075.2	37.15	184.73
23428	2707.5	2169.2	19.88	164.30
23429	2913.2	2087.4	28.35	140.01
a3429	561.5	310.7	44.67	115.13
23502	2428	1282.3	47.19	191.55
a3502	855.1	363.8	57.46	129.34
**Mean**	**1813.96**	**1179.81**	**36.68**	**153.38**
**SD**	**1159.73**	**841.13**	**13.02**	**35.51**
control DMSO 0.1%		current measured at end of 3-s pulse to +60 mV		
23503	6464.8	4281	33.78	180.19
a3503	1635.7	1239.6	24.22	140.54
a3509	2620.8	1707.8	34.84	131.61
23516	414.4	351	15.30	251.21
23525	1572.9	1196.9	23.90	144.26
a3525	718.4	647	9.94	126.79
**Mean**	**2237.83**	**1570.55**	**23.66**	**162.43**
**SD**	**2211.43**	**1411.01**	**9.86**	**47.39**
**Student's *t* test for independent samples (two-tailed)**			**p = 0.0639**<br>**t = 2.041 (12 *d.f.*)**	

Table S7. Summary of experimental results concerning the effects of cenobamate 200 µM on peak I_{Kr} amplitude measured during the second depolarizing step at -50 mV (R_a – access resistance).

**cenobamate 200 µM**		**peak current measured during step at -50 mV**		
**Exp. no.**	**I_{Kr} peak ini (pA)**	**I_{Kr} peak drug (pA)**	**% I_{Kr} inhibition**	**% R_a (fin/ini)**
a4627002	1244.28	1144.63	8.01	63.50
24628003	2443.19	2094.84	14.26	64.60
a4628003	2700.52	2351.44	12.93	98.63
24701003	568.12	526.56	7.32	67.46
a4701003	3402.70	2982.49	12.35	55.02
**Mean**	**2071.76**	**1819.99**	**10.97**	**69.84**
**SD**	**1145.68**	**979.60**	**3.11**	**16.75**

Table S8. Summary of experimental results concerning the effects of cenobamate 200 µM on AP parameters in amphotericin-perforated whole-cell patch-clamp experiments on Ncyte® vCardiomyocytes hiPSC-CM (R_a – access resistance).

Exp. no.	RP ini (mV)	RP ceno (mV)	APD90 ini (ms)	APD90 ceno (ms)	%APD90 change	%$I_{Na\ peak}$ change	%$I_{CaL\ peak}$ change	%I_f change	%R_a change
24917	-46.95	-39.4	1320	1135	14.02				100.00
a4917								64.34	-129.52
b4917	-50.32		1755			90.21	85.51	14.61	-170.63
24918	-47.06	-40.92	880.8	731.7	16.93	27.53	23.52	-22.97	43.40
a4918	-40.25	-48.49	678.7	471.7	30.50	-1.23	31.74		19.42
b4918									-43.80
24919	-65.46	-20.84	1857.7	242.8	*86.93	66.29	72.79	61.55	* -661.57
a4919									27.82
b4919	-57.59	-36.32	1246.1	820.6	34.15	-2.96	-5.90	-15.16	8.24
24920	-55.24	-40.34	711.2	274.5	*61.40	47.86			-5.55
a4920	-47.45	-54.14	1254.4	662.5	47.19	33.51	45.20	16.96	-144.26
b4920						-41.76	-6.72		50.02
c4920									100.00
**Mean**	**-51.29**	**-40.06**	**1212.99**	**619.83**	**28.55**	**27.43**	**35.16**	**19.89**	**-12.07**
**SD**	**7.84**	**10.44**	**442.61**	**317.12**	**13.50**	**42.15**	**35.68**	**36.91**	**91.70**

* Values excluded from statistics due to large depolarization, possibly seal destabilization.

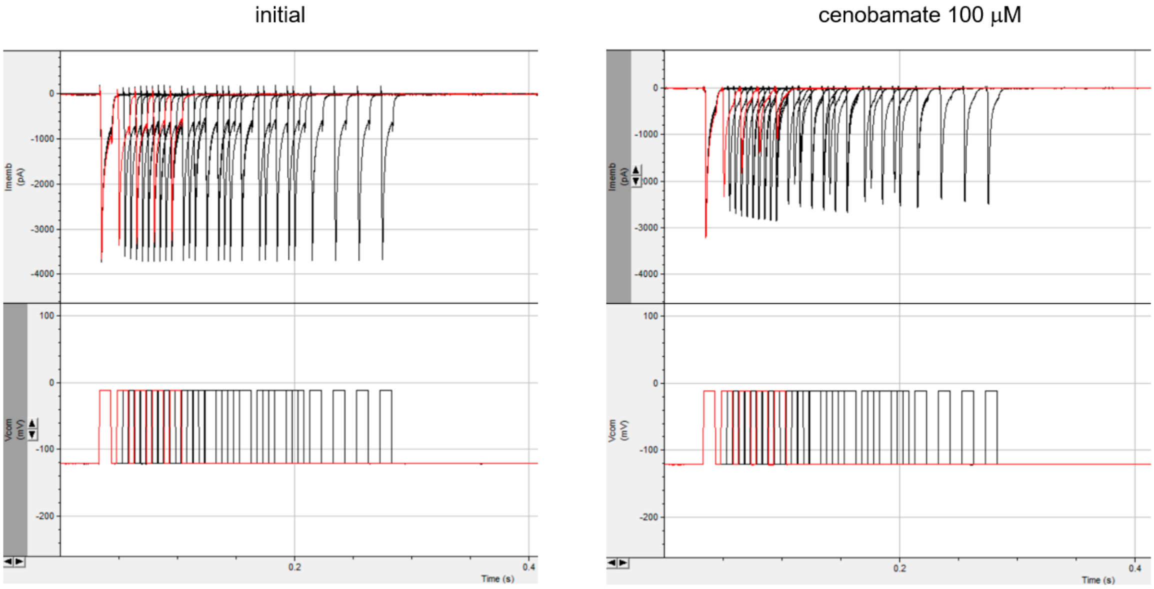

Figure S3. Voltage protocol Nav_freq_dep.pro for assessment of use-dependent block applied initially and with cenobamate in experiment c3o10.

S1. Analysis of variation of fraction of open channels during repeated depolarizing stimuli protocols used to assess use-dependent block

For this analysis we consider the Markov state diagram of Nav1.5 channels gating illustrated in Figure 5 of the main paper, including closed (C), open (O) and inactivated (I) states, the corresponding blocked states and some relevant transition rates. We note the probability of each state during the nth depolarizing pulse with non-capitalized symbols (e.g. o_n, i_n, ib_n, cb_n, etc.), the duration of the depolarizing pulse with t_d, and the duration of the subsequent repolarizing step with t_r. Thus, considering the initial probabilities of different states as cb_0, i_0 and ib_0, and neglecting blocking of open Na^+ channels during the first pulse until reaching peak amplitude, we obtain the peak open amplitude during the first pulse:

$$o_1 = 1 - cb_0 - i_0 - ib_0$$

We will first analyze the case of repeated depolarizing stimuli without drug application (the initial control recording), where only inactivation but not block by drug can occur. In this situation:

$$o_1 = 1 - i_0$$

that is the peak amplitude of the first Na^+ current transient depends only on the fraction of channels in steady-state inactivation at the holding potential preceding the series of pulses.

The fraction of inactivated channels at the end of the first depolarizing pulse with duration t_d will be:

$$i_1' = o_1(1 - e^{-t_d/\tau_i})$$

where τ_i is the (monoexponential approximation of the) inactivation time constant at the potential of the depolarizing step (average value 1.07 ms, see Table 2 of the main paper). During the repolarizing step of duration t_r, some of the inactivated channels will recover with time constant of recovery τ_r (again, in a monoexponential approximation), so at the end of the repolarizing step (and beginning of the next depolarizing pulse) the fraction of inactivated channels will be:

$$i_1 = i_1' e^{-t_r/\tau_r} = o_1(1 - e^{-t_d/\tau_i})e^{-t_r/\tau_r}$$

and the fraction of channels available for the second opening will be:

$$o_2 = 1 - i_1 = 1 - o_1(1 - e^{-t_d/\tau_i})e^{-t_r/\tau_r}$$

We can thus build a recurrence formula to express the fraction of open channels during depolarizing pulse n starting from the fraction of open channels during the previous pulse:

$$o_n = 1 - o_{n-1}(1 - e^{-t_d/\tau_i})e^{-t_r/\tau_r}$$

This recurrence formula allows us to compute the time constant of recovery from inactivation τ_r from experimental datasets, given all other parameters are known (τ_i can be estimated by monoexponential fits of the inactivating region of the current during the depolarizing pulse):

$$\tau_r = t_r / \left[\ln\left(1 - e^{-t_d/\tau_i}\right) - \ln\left(\frac{1 - o_n}{o_{n-1}}\right) \right]$$

We can write the entire series of recurrence relationships and devise a way to reduce equivalent terms in consecutive expressions:

$$o_{n-1} = 1 - o_{n-2}(1 - e^{-t_d/\tau_i})e^{-t_r/\tau_r}$$

which can be multiplied with $-(1 - e^{-t_d/\tau_i})e^{-t_r/\tau_r}$ and added to the previous formula expressing o_n. Thus we obtain the expression for the general term of the series:

$$o_n = \sum_{i=0}^{n-1} \left[-(1 - e^{-t_d/\tau_i})e^{-t_r/\tau_r} \right]^i - i_0 \left[-(1 - e^{-t_d/\tau_i})e^{-t_r/\tau_r} \right]^{n-1}$$

In obtaining these formulae we assumed that the potential of the depolarizing pulse is sufficiently positive to convert almost all open channels to an inactivated state, while the potential of the repolarizing step is sufficiently negative to remove almost all channels from inactivation; the assumption is valid for the combination of voltages -10 mV and -120 mV, as used in experiments (see Figure 2a of the main paper).

In the presence of blocker the recurrence formulae become even more complicated, because part of the inactivated channels will become blocked inactivated (*ib*) with the time constant τ_{ib} (the inverse of the sum of blocking and unblocking rate from the inactivated state), which we do not know.

We can assume a mixed "inactivated and/or blocked" state of probability *i+b* and we can assume that the inactivation rate only gets higher by adding the blocking rate:

$$(i + b)_1' = o_1(1 - e^{-t_d(1/\tau_i + 1/\tau_{ob})})$$

and at the end of the repolarization step of the first pulse:

$$(i+b)_1 = o_1(1-e^{-t_d(1/\tau_i+1/\tau_{ob})})e^{-t_r(1/\tau_r-1/\tau_{ib})}$$

Thus we can generate an equivalent recurrence formula and use it to fit experimental data to get the only unknown term, τ_{ib} :

$$o_n = \sum_{i=0}^{n-1}\left[-(1-e^{-t_d(1/\tau_i+1/\tau_{ob})})e^{-t_r(1/\tau_r-1/\tau_{ib})}\right]^i - i_0\left[-(1-e^{-t_d(1/\tau_i+1/\tau_{ob})})e^{-t_r(1/\tau_r-1/\tau_{ib})}\right]^{n-1}$$

It should be nevertheless much easier to use the fractional inhibition of peak current at step *n* relative to the peak amplitude at the previous step, which should be a constant:

$$(1-o_n)/o_{n-1} = (1-e^{-t_d(1/\tau_i+1/\tau_{ob})})e^{-t_r(1/\tau_r-1/\tau_{ib})}$$

Like in the case of control experiments, in the absence of blocker, this recurrence formula may be used to compute the time constant of inactivated channels block:

$$\tau_{ib} = 1/\left(1/\tau_r - \left[\ln\left(1-e^{-t_d(1/\tau_i+1/\tau_{ob})}\right) - \ln\left(\frac{1-o_n}{o_{n-1}}\right)\right]/t_r\right)$$

Of course, these formulae include a number of approximations, such as considering inactivation, recovery from inactivation and blocking/unblocking as processes with monoexponential kinetics, neglecting block of channels in the closed conformation due to very low affinity for this state, and neglecting the block of open channels since the start of a depolarizing pulse to the peak of Na^+ current amplitude – but we consider the errors introduced by these simplifying assumptions are in a manageable range.

S2. Modification of the O'Hara-Rudy 2011 model adapted for pharmacology simulations to include inhibition and data state-dependent blocking/unblocking of Na^+ channels by cenobamate

The initial C++ script of the model is described in Thomet *et al.* 2021 [1] and can be retrieved on on GitHub at https://github.com/bamuzesc/Qnet-for-CiPA.git and on Zenodo at https://zenodo.org/record/5615548#.YXvbQ7hyGSp.

We introduced a line to define inhibition data specific for cenobamate (concentrations are in nM, unblocking rates in ms^{-1}, blocking rates in $nM^{-1}ms^{-1}$):

```
if (cpd=="cenobamate")
{Cmax=170000;IKrIC50=1869000;IKrnHill=1; INaLIC50=46492.85;INaLnHill=1;ICaLIC50=509746.81;ICaLnHill=1;INaIC50=87557.97;INanHill=1;ItoIC50=100000000;ItonHill=1;IK1IC50=100000000;IK1nHill=1;IKsIC50=1336098.3;IKsnHill=1;IKrKf=1;IKrKu=1;IKrhalfmax=1;IKrnH=1;IKrVhalf=0;IKrkb=0.0000000002675;IKrkub=0.0005;actshift=0;}
```

In the definition block "Pharmacodynamic data" we included variables for the blocked state of Na^+ channels, its finite difference, and the state-specific blocking/unblocking rates of Na^+ channels:

```
//pharmacodynamic data
…
double b; //blocked state of INa by cenobamate
double db; //finite difference change in blocked state of INa by cenobamate
double kob=0.00000215; //nM^-1*ms^-1 state-dependent INa blocking/unblocking rates by cenobamate
double kobinv=0.189; //ms^-1 state-dependent INa blocking/unblocking rates by cenobamate
double kib=0.0000006698; //nM^-1*ms^-1 state-dependent INa blocking/unblocking rates by cenobamate
double kibinv=0.18252; //ms^-1 state-dependent INa blocking/unblocking rates by cenobamate
```

Further, we modified the RGC subroutine by defining specific equations for I_{Na} in the presence of cenobamate:

```
void RGC()
```

```
{
…
double GNa=75*scgna;  //orig GNa=75
double fINap=(1.0/(1.0+KmCaMK/CaMKa));
if (cpd=="cenobamate")
{
db=(m*m*m*((1.0-fINap)*h*j+fINap*hp*jp)*kob+m*m*m*(1-((1.0-
fINap)*h*j+fINap*hp*jp))*kib)*nCmax*Cmax*(1-b)-(m*m*m*((1.0-
fINap)*h*j+fINap*hp*jp)*kobinv+m*m*m*(1-((1.0-fINap)*h*j+fINap*hp*jp))*kibinv)*b;
b=b+db*dt;
 if (b>=1)
        b=1;
 if (b<=0)
        b=0;
INa=(1-b)*GNa*(v-ENa)*m*m*m*((1.0-fINap)*h*j+fINap*hp*jp);
}

else
{
INa=(1/(1+pow(((nCmax*Cmax)/INaIC50),INanHill)))*GNa*(v-ENa)*m*m*m*((1.0-
fINap)*h*j+fINap*hp*jp);
}
…
```

S3. Preliminary estimate of cenobamate effect on ventricular conduction velocity and the risk of induction of reentry arrhythmia in pathological myocardium with large conduction velocity heterogeneity

Given the large inhibitory effect of cenobamate on the cardiac-specific isoform of voltage-dependent Na^+ channels (Nav1.5), with an average inhibition of 69.5% ± 16.6% at 200 μM, leading to an estimated IC50 of 87.8 μM, well within the therapeutic range (reported C_{max} – maximal effective therapeutic plasma concentration – ETPC – of 169.98 μM), it is expected for the drug at clinically relevant concentrations to induce a significant block of the myocardial Na^+ current (I_{Na}) and subsequently a slow-down of conduction velocity, like a typical class Ib antiarrhythmic.

Using elementary computations, we tried to assess the consequences of this blocking effect on ventricular myocardium conduction velocity. Thus, we considered a normal value of intermyocyte longitudinal gap junction resistance surface density in healthy tissue of 2 Ω cm^2 [2, 3], and a 5 to 10-fold higher value in pathological tissue. This is equivalent to a gap junction resistance per cardiomyocyte of R_j = 0.526 MΩ, considering a cylindrical geometry and the average dimensions of human ventricular cardiomyocytes used in the O'Hara-Rudy 2011 model [4] (radius 0.0011 cm, length 0.01 cm). The same model uses a cell capacitance C_m of $1.534 \cdot 10^{-4}$ μF (153.4 pF), while the membrane resistance R_m per cardiomyocyte was estimated from R_m = 4.3 kΩ cm^2 (Cooklin M *et al.* 1998, [2]) and the geometric cardiomyocyte area $A_{geo} = 0.767 \cdot 10^{-4}$ cm^2 (O'Hara-Rudy 2011 model, [4]), yielding R_m = 56 MΩ. The electrical circuit of a cardiomyocyte of resistance R_m and capacitance C_m charged by a neighboring cardiomyocyte via gap junction resistance R_j is identical to the equivalent electrical circuit of a whole-cell patch-clamp configuration with cell elements R_m and C_m and access (pipette) resistance R_a. We have analyzed previously the dynamic charging regime of such a circuit and proved that the time constant of the circuit is $\tau = \frac{C_m R_m R_a}{R_m + R_a}$, which represents the time constant of an RC circuit with the two resistances, R_m and R_a, in parallel. However, in the ventricular cardiomyocyte case, since R_j is much smaller that R_m, it will dominate the parallel equivalent resistance and can be used as a first approximation for time intervals computation. In

this frame, the cardiomyocyte-to-cardiomyocyte propagation latency problem can be formulated in terms of computing the time required to charge the membrane capacitance of the second cardiomyocyte to a threshold potential (around -60 mV) from a resting potential of -90 mV, when the preceding cardiomyocyte is fully depolarized (around +40 mV), plus the phase 0 duration of this cardiomyocyte. For the equivalent circuit charging latency we can use the first order differential equation:

$$v_m(t) = v_\infty - (v_\infty - v_0)\exp(-t/\tau)$$

with $v_m(t)$ = -60 mV, v_0 = -90 mV, v_∞ = +40 mV, $\tau \approx 1.534 \cdot 10^{-4}$ μF · 0.526 M Ω = 0.08 ms, yielding t = 0.02 ms (a short time indeed). But if the gap junction resistance would increase 5 to 10-fold, this time would also increase proportionally, to 0.1 ms or 0.2 ms, respectively.

On the other hand, the magnitude of I_{Na} would exert an effect on the other part of the propagation latency, that related to the duration of phase 0 (fast depolarization). During this phase, I_{Na} is the most significant ion current charging the membrane capacitance C_m, therefore we can state the approximate relationship

$C_m \frac{dV_m}{dt} \approx I_{Na}$, and the maximum phase 0 slope would be roughly proportional to I_{Na} amplitude. In normal myocardium, taking a maximum phase 0 slope dV_m/dt = 400 mV/ms, a depolarization from threshold to peak potential (-60 mV to +40 mV, 100 mV potential difference) would last no less than 0.25 ms. Combined with a 0.02 ms gap junction propagation latency, for a typical ventricular cardiomyocyte 100 μm in length this would result in an average propagation speed of 0.1 mm / 0.27 ms = 0.37 m/s, which is a realistic value (compared to the average measured speed of 1 m/s).

Considering now some strongly pathologically altered values, e.g. an I_{Na} of 0.3 times normal, as obtained experimentally for cenobamate 200 μM, the phase 0 slope would decrease and the depolarization time would increase proportionally to 0.83 ms. For a ten-fold increase in gap junction resistance the gap propagation latency would also increase to 0.2 ms, giving a total latency of 1.03 ms and a propagation speed of 0.097 m/s. On a parallel fast pathway with normal gap junction resistance, the propagation speed in the presence of cenobamate 200 μM would be 0.117 m/s. The speed difference between a normal conductance (fast) pathway and a pathological altered conduction (slow) pathway would be in these conditions 0.02 m/s. Thus, to achieve reentry conditions, a time difference between the fast and slow pathway equal to at least one refractory period (~300 ms) should be achieved, resulting in a minimum length of the reentry circuit of 0.3 s· 0.02 m/s = 0.006 m or 6 mm, which is a realistic value. In the absence of cenobamate, the speed difference between the fast and slow pathways (again, with a ten-fold increase in gap junction resistance on the slow pathological pathway leading to a speed of 0.1 mm / (0.25 ms + 0.2 ms) = 0.22 m/s) would be 0.37 m/s – 0.22 m/s = 0.15 m/s, and the minimal length of the reentry circuit for a 300 ms refractory period would be 0.3 s· 0.15 m/s = 0. 045 m or 4.5 cm, a significantly larger value.

In addition to specific lengths of the reentry circuit, the reentry condition from the slow to the fast pathway requires that the depolarization wave front propagated along the fast pathway finds the slow pathway still in refractory period, while the same wave front propagated along the slow pathway will find the fast pathway after expiry of the refractory period and prone to receive excitation. Thus, the initial pacing frequency and the corresponding interstimulus period should be in an interval between the action potential duration and refractory period on the fast pathway and the same sum on the slow pathway, establishing an upper and a lower bound on the time window for slow-to-fast reentry. In practice it is quite difficult to accurately estimate this reentry window, because refractoriness is markedly altered by pathological conditions such as myocardial ischemia [5]. Another reentry mechanism is unidirectional block of conduction along the slow pathway with retrograde propagation of a stimulus from the fast pathway, again requiring a quite narrow vulnerability window, as discussed in Rudy 1995 [6].

Table S9. Number of cells featuring a propagated AP in computations with a linear string of 50 ventricular cardiomyocytes simulated with a modified O'Hara-Rudy 2011 model with included pharmacology data, in different conditions (G_j – gap junction conductance; C_{max} – maximal effective therapeutic plasma concentration of cenobamate = 170 μM).

G_j (pS/pF)	N cells with AP over 0mV								
	0 x C_{max}	0.5 x C_{max}	1 x C_{max}	1.5 x C_{max}	2 x C_{max}	3 x C_{max}	4 x C_{max}	5 x C_{max}	6 x C_{max}
6000	50	50	50	50	50	50	50	50	50
4500	50	50	50	50	50	50	50	50	50
3300	50	50	50	50	50	50	50	50	50
2700	50	50	50	50	50	50	50	50	50
2100	50	50	50	50	50	50	50	50	50
1500	50	50	50	50	50	50	50	50	50
1000	50	50	50	50	50	50	50	39	32
800	50	50	50	50	50	50	31	25	21
700	50	50	50	50	50	34	25	20	16
600	50	50	50	47	40	26	19	15	13
500	50	50	43	37	32	20	15	12	10
400	50	40	33	29	25	15	11	9	8
300	41	29	25	21	19	11	8	7	6

Table S10. End-to-end AP propagation time in computations with a linear string of 50 ventricular cardiomyocytes simulated with a modified O'Hara-Rudy 2011 model with included pharmacology data, in different conditions (G_j – gap junction conductance; C_{max} – maximal effective therapeutic plasma concentration of cenobamate = 170 μM).

G_j (pS/pF)	Conduction time (ms)								
	0 x C_{max}	0.5 x C_{max}	1 x C_{max}	1.5 x C_{max}	2 x C_{max}	3 x C_{max}	4 x C_{max}	5 x C_{max}	6 x C_{max}
6000	14.7261	14.7655	14.7771	14.7822	14.784	14.7902	14.9176	15.4267	15.4557
4500	17.7616	17.8228	17.8475	17.8635	17.875	17.9092	18.1348	18.6402	18.7605
3300	20.9949	21.0422	21.1953	21.6326	21.8058	21.9257	21.9793	22.0089	22.0598
2700	24.012	24.0908	24.1233	24.2349	24.6998	24.9937	25.0706	25.1098	25.3066
2100	28.1884	28.6588	29.0463	29.1362	29.1829	29.3701	30.0905	30.1905	30.3778
1500	36.1628	36.4032	37.1758	37.3062	37.4603	38.3044	39.1647	39.4335	40.3506
1000	49.5272	50.6468	51.6063	52.5772	53.5671	55.8073	71.9995	NA	NA
800	59.7708	61.8679	63.8048	66.1055	74.7225	176.95	NA	NA	NA
700	67.0518	70.6522	74.4989	106.686	158.204	NA	NA	NA	NA
600	77.0758	83.9967	136.378	NA	NA	NA	NA	NA	NA
500	91.9625	155.871	NA	NA	NA	NA	NA	NA	NA
400	127.307	NA	NA	NA	NA	NA	NA	NA	NA
300	NA	NA	NA	NA	NA	NA	NA	NA	NA

NA—not available; the AP failed to propagate to the end of string.

References

1. Thomet, U., Amuzescu, B., Knott, T., Mann, S.A., Mubagwa, K. & Radu, B.M. (2021). Assessment of proarrhythmogenic risk for chloroquine and hydroxychloroquine using the CiPA concept. *Eur J Pharmacol*, *913*, 174632. https://doi.org/10.1016/j.ejphar.2021.174632
2. Cooklin, M., Wallis, W.R., Sheridan, D.J. & Fry, C.H. (1998). Conduction velocity and gap junction resistance in hypertrophied, hypoxic guinea-pig left ventricular myocardium. *Exp Physiol*, *83*(6), 763-770. https://doi.org/10.1113/expphysiol.1998.sp004157
3. Rudy, Y. & Quan, W.L. (1987). A model study of the effects of the discrete cellular structure on electrical propagation in cardiac tissue. *Circ Res*, *61*(6), 815-823. https://doi.org/10.1161/01.res.61.6.815
4. O'Hara, T., Virág, L., Varró, A. & Rudy, Y. (2011). Simulation of the undiseased human cardiac ventricular action potential: model formulation and experimental validation. *PLoS Comput Biol*, *7*(5), e1002061. https://doi.org/10.1371/journal.pcbi.1002061
5. Kléber, A.G. & Rudy, Y. (2004). Basic mechanisms of cardiac impulse propagation and associated arrhythmias. *Physiol Rev*, *84*(2), 431-488. https://doi.org/10.1152/physrev.00025.2003
6. Rudy, Y. (1995). Reentry: insights from theoretical simulations in a fixed pathway. *J Cardiovasc Electrophysiol*, *6*(4), 294-312. https://doi.org/10.1111/j.1540-8167.1995.tb00402.x
